# Electrophysiology Read-Out Tools for Brain-on-Chip Biotechnology

**DOI:** 10.3390/mi12020124

**Published:** 2021-01-24

**Authors:** Csaba Forro, Davide Caron, Gian Nicola Angotzi, Vincenzo Gallo, Luca Berdondini, Francesca Santoro, Gemma Palazzolo, Gabriella Panuccio

**Affiliations:** 1 Tissue Electronics, Fondazione Istituto Italiano di Tecnologia, Largo Barsanti e Matteucci, 53-80125 Naples, Italy; csaba.forro@iit.it (C.F.); francesca.santoro@iit.it (F.S.); 2 Department of Chemistry, Stanford University, Stanford, CA 94305, USA; 3 Enhanced Regenerative Medicine, Fondazione Istituto Italiano di Tecnologia, Via Morego, 30-16163 Genova, Italy; davide.caron@iit.it (D.C.); vincenzo.gallo@iit.it (V.G.); 4 Microtechnology for Neuroelectronics, Fondazione Istituto Italiano di Tecnologia, Via Morego, 30-16163 Genova, Italy; giannicola.angotzi@iit.it (G.N.A.); luca.berdondini@iit.it (L.B.)

**Keywords:** 3D neuronal cultures, tissue engineering, brain-on-chip, brain organoid, neural spheroid, biomimetic tissue, in vitro electrophysiology, MEMS, microfluidics, biohybrid

## Abstract

Brain-on-Chip (BoC) biotechnology is emerging as a promising tool for biomedical and pharmaceutical research applied to the neurosciences. At the convergence between lab-on-chip and cell biology, BoC couples in vitro three-dimensional brain-like systems to an engineered microfluidics platform designed to provide an in vivo-like extrinsic microenvironment with the aim of replicating tissue- or organ-level physiological functions. BoC therefore offers the advantage of an in vitro reproduction of brain structures that is more faithful to the native correlate than what is obtained with conventional cell culture techniques. As brain function ultimately results in the generation of electrical signals, electrophysiology techniques are paramount for studying brain activity in health and disease. However, as BoC is still in its infancy, the availability of combined BoC–electrophysiology platforms is still limited. Here, we summarize the available biological substrates for BoC, starting with a historical perspective. We then describe the available tools enabling BoC electrophysiology studies, detailing their fabrication process and technical features, along with their advantages and limitations. We discuss the current and future applications of BoC electrophysiology, also expanding to complementary approaches. We conclude with an evaluation of the potential translational applications and prospective technology developments.

## 1. Introduction

Neurological disorders carry the highest global burden of disease [1]. Currently, they affect one billion people worldwide, and their impact is expected to increase due to the prolongation of life expectancy, along with other favoring factors such as environmental agents and lifestyle conduct. Therefore, there is an increasing need for reliable high-throughput models to address the mechanisms of brain development, function and dysfunction, and, ultimately, design and validate personalized medicine strategies. However, as the brain is the most complex organ of the human body, from the cellular, structural, architectural and functional standpoints, achieving biological models that can faithfully reproduce its complex features remains a long-standing challenge. Along with the highly heterogeneous cellular composition, the microenvironment, which is crucial for cellular metabolism and inter-cellular communication, is to be considered as an integral and active component of the brain. In this regard, there is a growing interest in developing strategies to mimic a realistic microenvironment in in vitro models of brain networks.

Brain-on-Chip (BoC) biotechnology represents the new frontier for modeling and studying the brain in a more realistic setting [2,3]. By combining three-dimensional (3D) brain-like systems with engineered microfluidics platforms, BoC aims at reproducing the brain architecture while providing an in vivo-like extrinsic microenvironment to the 3D cellular component. Moreover, methods from tissue bioengineering enable to enrich the 3D cellular construct with biomimetic biopolymers that mimic the extracellular matrix (ECM), a crucial component to maintain the intrinsic tissue microenvironment.

Currently, BoC finds its primary prospective applications in disease modeling, drug discovery, and personalized medicine. These range from understanding brain pathophysiology and pinpointing its underlying mechanisms, to the design and testing of new (personalized) treatments, including drugs and the so-called *electroceuticals* (i.e., treatments based on electrical stimulation). In addition, BoC holds promise to become the future mainstream biotechnology of regenerative medicine for the brain. In this regard, a holistic approach for studying the brain and possible novel therapies for its disorders should not only embrace cellular and molecular aspects, but also, and perhaps most importantly, the evaluation of its electrical function by means of electrophysiology. Such a 360-degrees approach becomes particularly relevant within the BoC biotechnology field in light of its relatively young age, for which an optimized and validated unifying framework in BoC design is likely to require a long-term research effort. While the field is primarily focusing on the development of microfluidics platforms and of more realistic 3D brain-like constructs, electrophysiology read-out tools are not yet routinely employed in BoC.

Here, we present an extensive review of the electrophysiology tools that could be coupled to BoC technology, describing their fabrication and discussing their advantages and limitations. This review intends to provide a solid foundation and a reference to researchers working in the BoC field, in the perspective of electrophysiology tools becoming a standard integration of BoC platforms and the mainstream technique for functional evaluation studies based on BoC.

## 2. Brain-on-Chip Biotechnology: A Historical Overview

BoC biotechnology has emerged as the result of the convergence of several disciplines, which, in parallel, have developed apparently unrelated technologies and biological approaches that are now core enabling technologies and methodologies for this flourishing field. These disciplines can be macroscopically categorized into two main pillars: (1) cellular biology and (2) microsystems technology. While the former has laid the foundation for advanced cell culturing techniques aimed at reproducing organs and tissues, the latter has brought microelectromechanical systems (MEMS) and microfluidics devices which today make up the artificial component of BoC.

### 2.1. Brief History of Bio-MEMS for Brain-on-Chip Biotechnology

MEMS are miniaturized systems integrating mechanical and electrical components. Their invention is owed to Harvey C. Nathanson, who, in 1965, described the first resonant gate transistor [4]. The device built upon the introduction of photolithography techniques in the 1950s to yield miniaturized printed integrated circuits. At the end of the 1970s, the integration of MEMS with microfluidics represented a milestone for the development of the first lab-on-chip platform [5]. Two decades later, the pioneering work by Manz [6,7,8] led to the development of the first micro total analysis system (μTAS) for automated chemical analysis of samples. His work has sparked outstanding advancement in microsystems technology, thanks to which it is nowadays possible to pursue high-throughput analysis in the fields of genomics, proteomics and metabolomics, with a high impact for biotechnology research, diagnostics, and medicine. Parallel to the introduction of μTAS, Whitesides’ team first described the use of soft lithography based on poly-dimethylsiloxane (PDMS) for rapid prototyping of microfluidics systems [9]. These pioneering works in MEMS and microfluidics have brought modern MEMS for biomedical applications (also referred to as biomedical- or bio-MEMS [10,11]), which represent today the core enabling technology for BoC [12].

**Figure 1:**
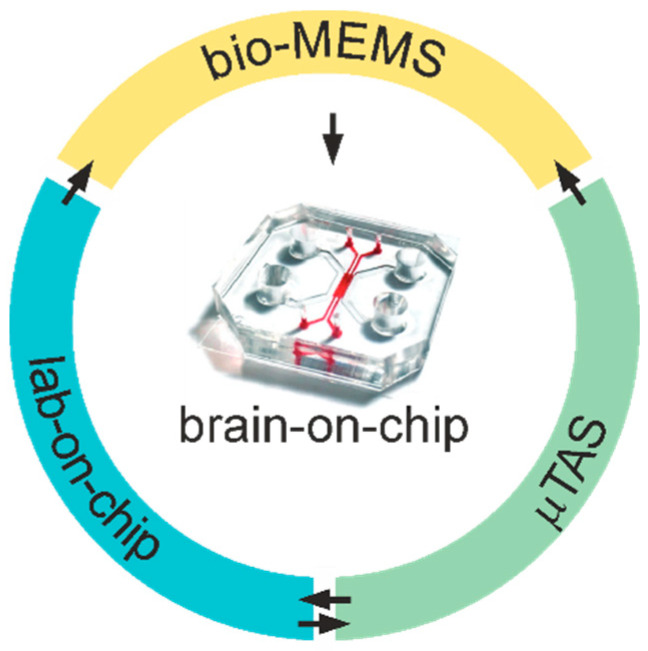
Brain-on-Chip biotechnology at the convergence of cellular biology and microsystems. Schematic diagram illustrating the mutual relationship between lab-on-chip and micro total analysis system (μTAS) technologies and their convergence into bio-microelectromechanical systems (MEMS) for Brain-on-Chip (BoC) biotechnology. The image of microfluidics chip at the center of the diagram is adapted from [13] with the permission of the Royal Society of Chemistry.

Bio-MEMS are combined MEMS-microfluidics platforms, which encompass several features of lab-on-chip and μTAS, such as biosensors and actuators. As detailed in §3.2, these features make bio-MEMS ideally suited for tissue engineering studies, therein including microfluidic and micro-patterned 2D cell cultures up to the more complex 3D BoC paradigm (Figure 1).

### 2.2. History of Cultured Neural Networks: From Cellular Monolayers to Bioengineered Brain Tissue

In the late nineteenth century, Wilhelm Roux, using cells of the neural plate of chick embryos, demonstrated that living cells can be successfully maintained in saline buffer [14]. Although the cells survived for only a few days, his early demonstration inspired further work to make in vitro cell culturing routinely possible. At the beginning of the twentieth century, Ross Harrison adapted microbiology culture methods developed by R. Koch to culture frog embryonic tissue known to give origin to nerve fibers so to observe nerve outgrowth in vitro. The developed technique, referred to as *hanging drop* [15], is now regarded as the very first in vitro cell culturing technique. Since then, there has been much progress in cell culturing, leading to improved and standardized protocols, which are now widely employed.

Two-dimensional (2D) neuronal cultures have represented, for a very long time, a valuable in vitro model to study neuronal network function and dysfunction, from the electrophysiological and molecular standpoints, as well as to assess the efficacy and safety of prospective therapeutic molecules. Their potential has been further expanded by the establishment of co-cultures, where multiple cell types are cultured together with the use of semi-permeable membrane inserts. Neuronal co-cultures allow investigation of the interplay between neurons and other different cell types, such as astrocytes [16,17], microglia [18,19], or both in the so-called *tri-culture* system [20], up to more complex heterogeneous cultures such as those including neurons, glia, endothelium and glioma cells [21], or those addressing multi-cellular tissue units, like neurogenic niches [22,23] and the blood–brain barrier (BBB) [24].

**Figure 2:**
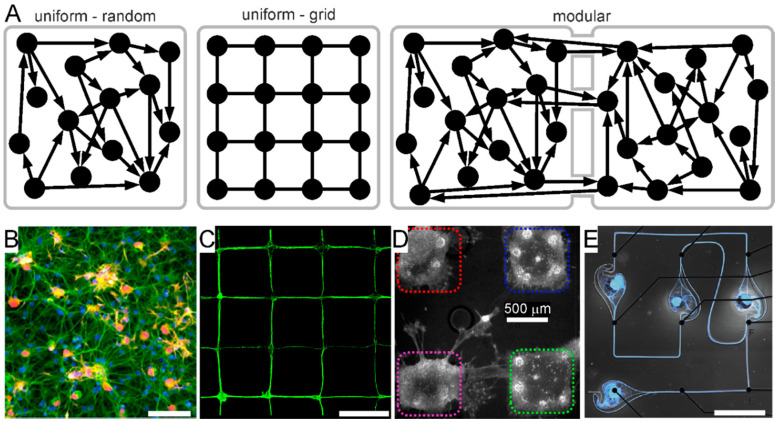
2D neuronal network topologies obtained with network engineering techniques. (**A**) Schematic diagrams of different network topologies obtained with and without the use of network engineering techniques. Uniform random cultures are made of uniformly distributed neurons connecting in a random network architecture, i.e., without a preferential site of adhesion, directionality or connectivity. Uniform neuronal networks with an imposed topology can be obtained via bio-printing and patterning techniques, dictating the site of cell adhesion as well as the path of neuronal processes outgrowth. In vitro modular neuronal network models can also be defined by two or more random modules, where the connectivity between/among modules is the sole topology parameter controlled by the bioengineering strategy. (**B**) Microscopy image of a uniform random culture of primary hippocampal neurons at 21 days in vitro. Scale bar: 100 µm. Adapted with permission from [26]. Copyright © 2014 Elsevier B.V. (**C**) Fluorescence microscopy image of a uniform grid engineered neuronal network obtained with combined micro-contact printing of an adhesion promoter and coating of a repulsive (agarose) layer. Scale bar: 200 µm. Adapted with permission from [31]. (**D**) Multi-modular primary neuronal networks obtained with poly-dimethylsiloxan (PDMS) mask-guided bioprinting, showing inter-module connections. Adapted with permission from [42]. (**E**) Engineered interconnected neuronal microcircuits matching the spatial distribution of microelectrodes within an electrode array. The microcircuits were obtained by directional geometric guidance provided by PDMS microstructures. Scale bar: 200 µm. Adapted with permission from [33].

Most commonly, 2D neuronal cultures are uniform (Figure 2A,B), wherein neurons are evenly distributed on the culturing substrate, and the network architecture is typically random, i.e., without a preferential site of adhesion, directionality or connectivity [25,26]. Such characteristics imply a certain degree of variability among cultures and, most importantly, do not realistically represent brain networks organization that is the signature of the brain.

In search for a more realistic representation of brain networks topology, at the end of the twentieth century, research started focusing on strategies to obtain compartmentalized neuronal cultures that would enable addressing both structure and function of neuronal networks in vitro. The first chambers for neuron compartmentalization were developed by Campenot in the 1970s using machined Teflon dividers to provide spatial and fluidic separation of distal axons from the soma of long-projecting neurons [27,28]. At the end of the 1980s, Klenfield and colleagues were the first to obtain patterned neuronal cultures by implementing a combined surface chemistry–photolithography approach [29,30]. Their seminal work marked the inception of network engineering. Building upon their work, researchers have started to further develop soft lithography techniques brought by Whitesides to implement (bio) patterning approaches, such as micro-contact printing and microstructures as physical barriers [31,32,33,34,35,36,37,38]. Along with more recent guidance techniques based on functionalized vertical nanopillars [39], these approaches enable the generation of patterned (uniform grid or modular) 2D neuronal networks of defined topology at cellular [31,34,35,36,39,40] or population scales [25,31,38,41,42,43,44,45] (Figure 2C–E). These strategies have enabled investigation of the interplay between anatomical connectivity and dynamics in neural networks in a reductionist and simplified approach. Electrophysiology and modeling studies have indeed evidenced that modular neuronal networks exhibit different activity dynamics as compared to their uniform counterpart [25,44,45,46,47,48]. These different culture topologies therefore enable addressing different features of brain networks, such as synaptic scaling, signal propagation, connection directionality, and the interaction among sub-networks. Moreover, in the perspective of translational studies, modular neuronal networks were also proposed to model the functional interplay among several brain regions, as well as brain lesions (by disconnecting one or more modules) in a simplified and accessible setting [49]. These approaches not only may provide useful insights into the functional role served by specific brain structures (brain physiology), but also about the re-arrangement of damaged brain networks (brain pathophysiology), and, ultimately aid in the design of neuroprosthetics devices [50] and regenerative approaches aimed at re-establishing lost connections between neuronal networks across brain areas. In this view, the modular culture strategy may be implemented using neurons derived from different brain regions, which are known to be connected in the brain, as recently explored in the 2D multi-regional [51] and *assembloid* [52,53,54] BoC paradigms (*cf.* §3.1.2).

Nonetheless, 2D culture systems, whether random, patterned, or multi-modular, lack the third dimension and the supportive microenvironment provided by the ECM typical of native biological tissues. This affects primarily cell polarization and morphology and, consequently, cell functions, as physical and mechanical constraints influence cell mechanotransduction, i.e., the activation of biochemical pathways upon external forces [55]. Given the high correlation between structure and function in body organs, the alterations occurring at the cellular and molecular levels are reflected at the organ level, for which 2D culture systems are known to miss key in vivo functional hallmarks of the organ of origin. The tight structure–function relationship is particularly relevant in the brain, which coordinates the activity of other organs and systems and serves higher cognitive functions.

The limited geometry of 2D culture systems has been progressively surpassed by brain tissue bioengineering, starting with the seminal work performed at the end of the twentieth century by Elsdale and Bard, who described the first method for three-dimensional (3D) culturing techniques based on collagen substrates [56]. Three decades later, Alexis Carrel reported on the cultivation of explanted tissues [57,58]. His work, inspired by the technique set up by Harrison, was the first one to mention the use of what we would consider today a scaffold biomaterial, i.e., silk fibers as a structural support to prevent disaggregation of the cultured tissue. These studies marked the inception of tissue bioengineering and supported progress in regenerative medicine.

## 3. Methods for Generating Brain-on-Chip

Brain tissue bioengineering provides the core biological substrate for BoC. It combines neurons and non-neuronal cells, growth factors, small molecules and biomaterials with the aim of obtaining in vitro replicas of brain areas, mimicking their native anatomical features. These include the 3D geometry along with the layered organization (as opposed to 2D monolayer cultures), the physico-mechanical properties, the co-existence and intimate interaction between cells and ECM. The synergic blending of these key ingredients contributes to generate in vitro functional cellular assemblies that resemble, even though in a simplified manner, the structural and functional organization of brain tissue.

One fundamental aspect to consider for bioengineering a functional biological tissue is the microenvironment. This includes numerous factors, which can be grouped into two main categories: intrinsic and extrinsic.

The intrinsic microenvironment is the so-called extracellular space embracing the rich plethora of nutrients, growth factors, small molecules, and ions, which are embedded in the macromolecular net of the ECM. Hence, the ECM does not only represent the scaffold around which brain tissues take shape but it also provides important cues to guide cell fate, intercellular connections, synapse formation and signal transmission.

The extrinsic microenvironment is made up by the conditions external to the bioengineered tissue, which are necessary for its functions and self-maintenance, like oxygen level, temperature, humidity, and the medium perfusion conditions.

Here, we provide a classification of 3D neuronal/multicellular cultures based on the methods used for obtaining them. We also describe the currently available strategies to reproduce permissive intrinsic microenvironments, as well as the use of MEMS/microfluidics to maintain an appropriate extrinsic microenvironment.

### 3.1. Available Biological Substrates

Many research groups make an indifferent use of 2D or 3D cell cultures as biological substrate for BoC. However, here, we narrow our description to 3D bioengineered cultures, as the most relevant and the most responding to the definition of BoC (*cf.* §1). In this regard, a first distinction should be made about the cell source used to obtain bioengineered brain tissue, which can be classified into (i) primary cells, (ii) immortalized cell lines, and (iii) stem cells.

Primary cells, including mature neurons, astrocytes, microglia or oligodendrocytes, are freshly isolated from mammalian brain tissue and can be used for establishing short-term (few weeks) in vitro cultures [59]. Immortalized cell lines, such as SH-SY5Y [60,61] and PC12 [62], are generated from either healthy or tumor tissue, in order to obtain cells that can indefinitely grow in vitro. Immortalized cell lines thus enable highly reproducible and well-characterized biological substrates for long-term in vitro studies. Stem cells are undifferentiated cells endowed with potency, i.e., the ability to differentiate in different cell types. The discovery of stem cells and their potency has revolutionized the way of thinking about experimental cell cultures and paved the way for tissue engineering and regenerative medicine [63,64]. Stem cells can be obtained from embryos at early stages of development. In this phase, the cells of the inner cell mass, also known as embryonic stem cells (ESCs), are identical and pluripotent, i.e., able to differentiate in nearly all cell types [65,66]. Alternatively, embryos at later stages of differentiation and adult tissue are an important source of special niches of multipotent stem cells able to yield cell types specific of the tissue they were extracted from. Adult neurogenic niches populated by neural stem cells (NSCs), first described by Altman and Dan in 1965 [67], are nowadays an important biological means to study brain development and regeneration [68]. The horizon of stem cell research and its potential applications has been greatly expanded since the ground-breaking discovery of Takahashi and Yamanaka [69] that adult differentiated cells can be genetically engineered to revert their phenotype to undifferentiated pluripotent proliferating cells (induced pluripotent stem cells—iPSCs).

#### 3.1.1. Scaffold-Based 3D Neuronal Cultures

Scaffold-based 3D cultures are built with the use of biomaterials that provide the bearing structural frame for the embedded cells, permitting their vertical stacking and distribution across the three dimensions. Scaffold-based 3D cultures have been developed by the avant-garde research of S. Przyborski [70,71], which has inspired subsequent work to advance research in scaffolding biomaterials for tissue bioengineering.

Scaffold-based 3D cultures typically offer a better spatial control and more physiological behavior than conventional 2D systems. Indeed, the 3D architecture of scaffold-based cultures has been correlated to specific features of network dynamics like the simultaneous presence of local and global activity patterns and their spatiotemporal regulation, which resemble what observed in the mammalian brain [72,73].

The scaffold can be generated from a wide variety of biomaterials, which can be natural of various origin (i.e., animal, vegetal, bacterial), synthetic, or semi-synthetic (Table 1), whose physico-mechanical properties can be virtually adapted to any target tissue [74]. Indeed, the scaffold properties, such as porosity, stiffness, and toughness, influence important cell functions including proliferation, migration, differentiation and tissue formation [75]. Their key role in guiding axonal extension and synapse formation has been especially demonstrated in the absence of bioadhesive cues [76]. The prospect of fine-tuning the physico-mechanical properties of the scaffold makes it particularly appealing for reproducing extremely soft tissues like the brain. In addition, scaffolds are suitable for investigating cell response to physico-mechanical stimuli mimicking brain viscoelasticity perturbations following concussions or disease progression, which are in turn reflected in altered activity patterns [77].

**Figure 3:**
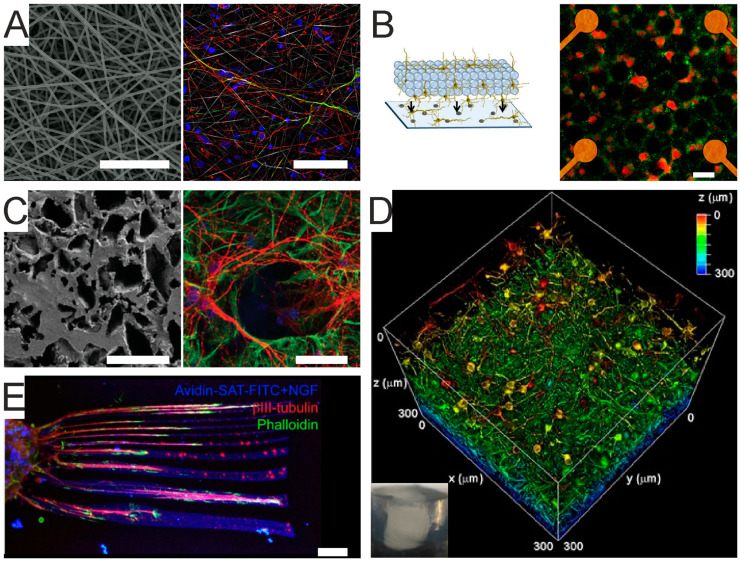
Scaffold-based 3D cultures. (**A**) Electrospun fibers scaffold. Left—Scanning electron microscopy of thick electrospun fibers generated from tyrosine-derived polycarbonates. Scale bar 100 mm. Right—reprogramming induced pluripotent stem cells (iPSCs) on 3D electrospun fibers, leading to the generation of bIII-tubulin+ (red) and MAP2+ (green) neurons. Scale bar: 50 mm. Adapted from [79]. (**B**) Microbeads scaffold. Left—multilayered assembly of microbeads and primary neurons coupled with 2D primary neuronal cultures grown on a microelectrode array (MEA). Right - immunostaining of 3D culture on MEA, showing MAP-2+ (green) and NeuN+ (red) neurons. Scale bar: 40 μm. Adapted from [83]. Copyright © 2014 The Authors. (**C**) Graphene scaffold. Left—scanning electron microscopy image of a nanostructured PDMS–graphene scaffold. Scale bar: 200 m. Right—primary hippocampal neurons at 10 day-culture within the scaffold (green, betaIII tubulin+ neurons; red, GFAP+ glial cells). Scale bar: 50 μm. Adapted from [80]. Copyright © 2020 The Authors. (**D**) Alginate hydrogel scaffold. 3D reconstruction of a 300-μm^3^ volume of a cortical culture at 53 days in vitro. Color bar indicates the color-coded depth. The inset shows a macroscopic view of the bulk homogeneous alginate hydrogel. Adapted from [97]. (**E**) Hyaluronic acid hydrogel scaffold in which chick dorsal root ganglia axons are elongating within two-photon patterned microchannels functionalized with nerve growth factor. The bio-functionalized microchannels enable axon guidance within the hydrogel. Scale bar: 50 m. Adapted with permission from [86].

Biomaterials can be tailored to achieve scaffolds of any architecture (Figure 3), including highly porous solids [78,92], electrospun fibers [79], micro- or nano-structures [73,83,98], bulk homogeneous [76,85,94] or patterned hydrogels [86].

Solid scaffolds can provide a high degree of porosity and thus a high surface area for cell adhesion and cell–cell connections, yet they are relatively poorly tunable in terms of mechanical properties and optical transparency [78,92]. Electrospun fibers (Figure 3A) are highly controllable in terms of fiber thickness, geometry and interspaces, and have been successfully used to regulate stem cells’ differentiation and maturation into functional neurons [79]. Microstructured scaffolds can be obtained by stacking, layer by layer, adhesive microbeads (Figure 3B). The inter-bead spaces can accommodate neurons (or any cell type) and allow axons/dendrites to extend through the layers, thus enabling neuronal connections, which, in turn, give rise to complex network activity patterns [83,98].

Nanostructured scaffolds (Figure 3C) are obtained by endowing polymeric structures (e.g., PDMS, poly-acrylamide) with nanomaterials like carbon nanotubes and/or graphene, thus inserting nano-topologies relevant to increasing cell–material interactions. Additionally, both carbon nanotubes and graphene are becoming particularly attractive in brain bioengineering due to their high inherent electrical conductivity and ability to facilitate tissue excitability, as supported by recent findings that these nanomaterials improve synaptic activity and network synchronization, likely influencing the balance between depolarizing and hyperpolarizing currents [73,80,82].

Hydrogels (Figure 3D) are particularly compliant scaffolds in that they are highly tunable in terms of physical–mechanical properties. Hydrogels can be of natural origin, obtained from mammalian and non-mammalian sources, or synthetic. Hydrogels of natural mammalian origin can be obtained as native ECM extracts (e.g., from decellularized human adipose tissue [104]) or can be polymers that are naturally abundant in the brain’s ECM, such as hyaluronic acid [85]. Hydrogels of natural non-mammalian sources are biomimetic biopolymers obtained from non-mammal animals, bacteria, or of vegetal origin (e.g., alginate [94,95,96,97], chitosan [87,88], silk fibroin [102]). Synthetic hydrogels are usually inert polymers (e.g., polyethylene glycol) functionalized to become bioactive and/or mixed with high molecular weight polymers (e.g., polysaccharides) to generate interpenetrating networks [76]. Hydrogel complexity can increase via tailoring with functional groups that exert bioadhesive, biodegradable or bio-instructive properties [76,90].

3D scaffolds can be either homogenous or patterned (Figure 3E). The latter are deployed to guide cell process elongation in specific directions and mimic cell orientation, which, in turn, promotes coordinated spiking activity as seen in native brain tissue. Patterning can be achieved via diverse approaches [105], including photo-chemical cross-linking [84], photo-ablation [84,91], and bioprinting [89,99,106,107]. 3D scaffolds are suitable to generate multiple co-cultures within the interconnected porous structure, promoting the development of more complex organizations of neuronal networks. Functional co-cultures are feasible in different kinds of scaffolds, including layered hydrogels where different cell types are distributed in adjacent layers to mimic tissue niches, such as the cortico–hippocampal interface [97] or the neural progenitor cell niche undergoing migration and differentiation towards mature neuronal phenotypes [77]. Paper-based 3D astrocyte cultures deposited on cortical neurons have been employed to investigate the cortical–astrocyte interface [93], whereas carbon nanotube nets developed within graphene foam scaffolds have been used to investigate invasiveness of glioma cells in tissue-like structures generated by cortical networks [81].

The most common technique used for functional evaluation of 3D scaffold-based cell cultures is calcium imaging (*cf.* §5.1), since it is quite an easy technique to implement. Only a few works report on electrophysiological recording from hydrogels, namely whole-cell patch-clamp recording [76,99,106] and extracellular local field potential recording using tungsten electrodes [101,102] or glass microelectrodes [101,103]. Network electrophysiology via microelectrode array (MEA) appears to be somewhat problematic. Possibly, the scaffold material that is inherently interposed between cells and microelectrodes acts as a shield, which hinders a good electrical coupling between cells and MEA. Specifically, it is possible that non-degradable scaffold materials contribute to this phenomenon, whereas degradable scaffolds may overcome this issue by allowing neurons and/or their extensions to reach the electrode surface. So far, only a few studies have reported the possibility of effectively coupling 3D scaffold-based neuronal cultures with MEA via different strategies, such as exploiting biodegradable hydrogels [100], coupling the 3D culture with 2D cultures that are in direct contact with the electrodes [83,98], or a combination of the two approaches [108]. It is worth noting that application of gentle pressure was sufficient to obtain good-quality MEA recordings from 3D cultures where the scaffold was an omentum-derived hydrogel [104]. An alternative approach to improve cell–microelectrode coupling is using paper-based 3D cultures, which offer the possibility of reducing the neuron–electrode distance. This strategy has been successfully used to investigate network activity and signal propagation in neuronal cultures [92] and in astrocyte–neuron co-cultures [93].

Overall, 3D scaffold-based cultures achieve a higher degree of neuronal maturation and more complex connectivity patterns, compared to 2D cultures. Hence, 3D cultures represent invaluable tools for exploring cellular and network behavior in a more natural setting and, if coupled with advanced technologies such as bioreactors, bioprinting and/or MEMS and microfluidics, they can reproduce, at even higher levels, the complexity of the full organ or tissue unit.

#### 3.1.2. Neural Spheroids and Organoids

Neural spheroids and organoids are cellular aggregates ranging in size from the micro- to millimeter scale, which differ in their formation process and in their architecture but share the common feature of being generated with similar strategies. In fact, both are obtained by providing either of intrinsic and extrinsic factors that promote cell aggregation, such as low adhesive substrates, biomimetic ECM, continuous stirring, conic wells and, in the most advanced bioengineering approaches, microfluidics and bioreactors [109].

**Figure 4:**
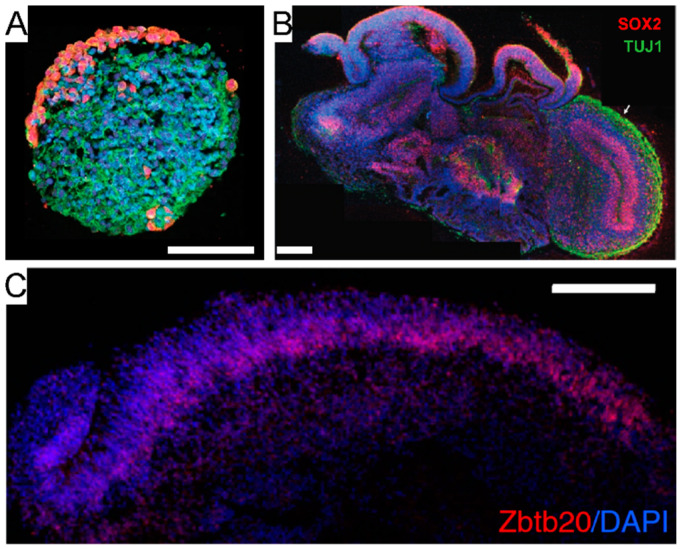
Spheroids and organoids. (**A**) Brain spheroid. Human iPSC-derived brain spheroid using primary glioblastoma cells, stained for glia (GFP, red) and neurons (Tuj1, green). Scale bar: 100 μm. Adapted with permission from [121]. Copyright © 2019, The Authors. (**B**) Whole-brain organoid. Sectioning and immunohistochemistry reveal a complex morphology made of heterogeneous regions, and the presence of neural progenitors (SOX2) and neurons (TUJ1, arrow). Scale bar: 200 μm. Adapted with permission from [124]. Copyright © 2013, Nature Publishing Group. (**C**) Region-specific organoid. Hippocampus-like tissue expressing the specific marker Zbtb2. DAPI: nuclei. Scale bar: 100 μm. Adapted with permission from [133]. Copyright © 2015, The Authors.

Spheroids are the simplest form of cellular aggregate, wherein the cells are randomly organized and tightly packed (Figure 4A). Despite the random architecture, cells within spheroids establish close intercellular interactions similar to native tissue, thanks to their density [110,111]. Spheroids can be made of either homogeneous or heterogeneous cell populations, represented by any cell type including primary cells [111], cell lines [112], and progenitor cells [113]. The latter provide neural spheroids with longer life and more functionalities in light of their higher self-renewal and differentiation capabilities [113]. For example, it has been shown that human-iPSCs-derived cortical spheroids are populated by excitatory neurons typical of the dorsal telencephalon and that their cytoarchitecture resembles the laminated neocortex including neurons expressing both deep and superficial-cortical layers markers [114], whereas another study has shown that spheroids can effectively recapitulate astrocyte maturation [115]. These models could be further implemented with microglia cells [116] or basic vascularization to resemble most of the in vivo features of the BBB [117].

Neural spheroids have also been obtained via a PDMS-based network stamping method [118], wherein a PDMS mask was used to create a micro-chamber array accommodating the spheroids as well as micro-channels to allow spheroid interconnections. Remarkably, the neurospheroid network could be successfully grafted onto the cortical surface of a rodent brain, after which the grafted neurons extended their axons and formed synaptic connections with the host cortical neurons, and their electrical activity persisted for ~1 week.

Neural spheroids show a high flexibility in recapitulating the pathological landscape in vitro, from monogenic alterations, such as tuberous sclerosis [119], to cancer, including glioblastoma multiforme [120,121] and medulloblastoma [122]. Indeed, spheroids are particularly useful for mimicking amorphous tissues, like tumors, due to their random structural organization [110,123]. Nonetheless, in light of their lack of cellular polarity and layered structure typical of brain tissue, they are not suitable for brain development studies or for studies requiring an architectural replica of specific brain areas. This drawback has been overcome by the advent of brain organoids, thanks to the pioneering work of M. Lancaster and colleagues [124].

Brain organoids are self-assembled cell aggregates in which cells are not just randomly organized and interacting with each other, but exhibit a high degree of organization that closely resembles the brain tissue polarity, for which the cell-to-cell interplay is spatially and temporally regulated [125,126]. These bioengineered tissues can either reflect brain structures at large, in which case they are referred to as brain or cerebral organoids (Figure 4B), or rather resemble specific brain regions, in which case they are referred to as region-specific organoids (Figure 4C), such as adenohypophysis [127], cerebellar [128], forebrain [129,130,131], midbrain [132], hippocampal [133], hypothalamic [134], choroid plexus [135], optic-cup [136], or retinal organoids [137].

In light of their exponential development over the past few years and the potential further development in the next decades, brain organoids are definitely the latest frontier of tissue bioengineering. Specifically, within the BoC biotechnology field, they are likely to soon become the mainstream approach to study brain development, function and dysfunction, as well as for addressing the efficacy and safety of potentially novel therapeutic treatments for brain disorders. Ultimately, they hold great potential to become the core biological substrate of regenerative approaches for the brain, as very recently heralded by the work of Mansour and colleagues [138] and of Dong and colleagues [139], who have demonstrated their suitability for grafting into the brain.

As the organoid (likewise the spheroid) generation is based on cell aggregation, it is expected that cell–cell interactions are highly promoted. In order to also establish cell–matrix interactions, especially in the initial phases of cellular aggregation, when cells have not yet secreted their own ECM, it is important to provide a surrogate ECM that mimics the native intrinsic microenvironment and guides tissue organization. Most of the studies have so far employed the commercially available matrigel to sustain organoid development [124,140,141,142,143,144]. However, due to its animal origin (derivation from murine sarcoma [145]), matrigel presents significant limitations, including fast degradation rate, not fully characterized composition, batch-to-batch variation, and poor translational potential. To overcome these issues, biomimetic biomaterials are starting to be employed to generate defined matrices and guide organoid development [146].

**Figure 5:**
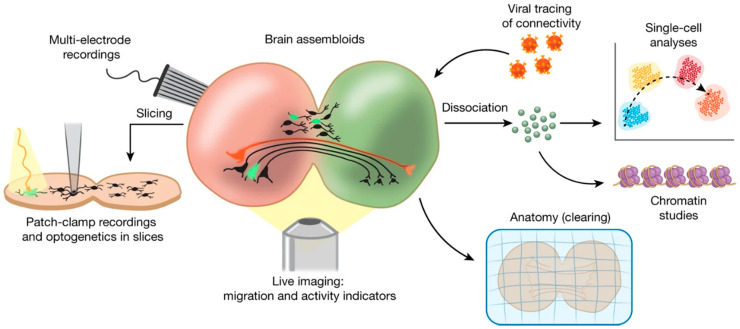
Assembloids. Brain assembloids developed by S. Pașca can be processed and analyzed using a plethora of techniques, including patch-clamp and multi-electrode extracellular electrophysiology recordings, single-cell analyses, transcriptomics and proteomics, chromatin studies, tissue transparency methods for 3D reconstructions, viral tracing for connectivity assessment (e.g., retrograde labelling), live imaging such as calcium and voltage biosensors, and optogenetics. From [53] with permission.

From the functional standpoint, electrophysiological studies have highlighted key features of brain organoids exhibiting inter-cell communications based on electrochemical signals passing through one or more physically connected circuitries. This is particularly evident in the so called *assembloids*, a term coined by S. Paşca to describe the anatomical and functional assembly of multiple organoids (or spheroids), wherein infiltrating nerve fiber branching among two or more masses mimics interconnected brain areas [52] (Figure 5). Assembloids can be processed and studied via a plethora of approaches like any other biological sample, thereby allowing full anatomical, functional, and genomic characterization studies. Moreover, they are a unique means to study the development of interconnections as well as cell migration among brain areas in a controlled setting [147]. Therefore, assembloids carry the tremendous potential of enabling the replication (and characterization) of interconnected brain areas at the highest level of complexity so far witnessed in the tissue bioengineering field [53,54].

Functional connections among different regions within brain organoids have also been recently reported in organoids maintained at the air–liquid interface [141], a strategy that seems to improve the functionality and interconnectivity of brain organoids. Remarkably, along with the reported ability of brain organoids to recapitulate brain development [124], a recent study has demonstrated that the spontaneous network activity generated by cortical organoids is reminiscent of brain patterns generated by the preterm human [148]. In keeping with this, it has been demonstrated that brain organoids generate giant depolarizing potentials (GDP), which progressively disappear during organoid maturation, and exhibit a parallel developmental polarity switch of the primary inhibitory neurotransmitter γ-aminobutyric acid (GABA), namely, from depolarizing to hyperpolarizing [149]. These two phenomena are inter-related and are known to occur in the developing brain [150] [151,152]. Nevertheless, brain organoids still show many limits as compared to the human brain, most of them due to a less broad spectrum of different cell types and a relevant presence of stress hallmarks that could affect in vitro organoid development [144]. In addition, current protocols for organoid development have not yet been able to achieve a degree of maturation comparable to the juvenile or even the adult brain, which would be desirable to better mimic the brain physiological and pathological mechanisms in adolescence and adulthood [153].

Functional analysis of brain spheroids and organoids most commonly relies on calcium imaging (*cf.* §5.1) as an overall screening technique of the tissue functionality [52,133,149,154,155]. As the field has started witnessing an exponential growth, in-depth characterization by means of electrophysiology studies has become more frequently employed in order to pinpoint the biophysical phenotype of the cell population, as well as network dynamics that cannot be resolved in detail by calcium imaging approaches. Table 2 summarizes on the used techniques and sample preparations for electrophysiology studies of brain organoid function.

Patch-clamp recording is so far the most commonly used electrophysiology technique and it has been used to address the presence of Na^+^ and K^+^ currents involved in action potential dynamics, the firing properties of neurons, as well as to address the presence of excitatory and inhibitory synaptic activity [52,133,148,154,156,157,158,159]. In this regard, the available information still remains qualitative, whereas a quantitative analysis as well as a direct comparison with the native brain structure of reference is yet to be completed.

MEA recoding is very popular to obtain network-wide information that not only allows detailed network dynamics studies of the 3D tissue *per se*, it also enables a direct comparison with the human brain electrical patterns [141,148,149,158,160,161]. It needs to be mentioned, however, that MEA recording of the intact spheroid or organoid using planar MEAs poses some difficulties in achieving a good electrical contact between the electrodes and the tissue, as the latter does not offer a flat surface that can optimally adhere to the planar MEA substrate. To overcome this issue, two main approaches may be considered to improve the yield of network electrophysiology assessment: (i) sample processing and (ii) recording device.

In terms of sample processing, one strategy consists of letting the intact tissue sit on the MEA, previously coated with adhesion molecules (poly-(D/L)-lysine, poly(L)-ornithine, polyethylenimine, laminin) for several days or weeks [148,158,160,161]. However, such a procedure is very likely to induce cell spreading and organoid disaggregation, for which the recorded signal might as well represent the result of secondary 2D network activity, wherein the 2D networks are established upon guidance by the coating biomolecule. The other strategy consists of slicing the tissue to obtain thin (200–300 μm) sections so to access the inner tissue layers and bypass its surface, which most frequently contains non electrically active stem cells [52,149]. These approaches do not fully preserve the original 3D circuitry in full, whereas, ideally, electrophysiological measurements should be performed on the intact tissue assembly in order to obtain a global picture of its network dynamics.

In terms of recording device, silicon array probes (*cf.* §4.3.2) inserted directly into the intact tissue sample [142,159] have been successfully employed to overcome the technical limitations posed by planar MEAs. Silicon probes enable recording local field potentials, as well as single- and multi-unit activity, with the added advantage of enabling a depth electrophysiology profile of the bioengineered brain tissue. 3D MEAs (*cf.* §4.3.1) may also represent a valid alternative, but so far, to the best of our knowledge, they have only been used to record from organoid slices and never from the intact organoid [141].

Finally, it is important to remark that the electrophysiological characterization of brain organoids remains very challenging due the high batch-to-batch variability, which results from the complex interactions between many intrinsic and extrinsic factors involved in the organoid development. As we progress towards a better understanding of the phenomena underlying in vitro tissue formation, it will be possible to generate brain organoids under more controlled and highly reproducible conditions, which, in turn, will facilitate a more in-depth functional analysis by means of electrophysiology techniques.

#### 3.1.3. Organotypic Cultures

Organotypic cultures are established from a tissue explant and maintained in culture for long periods. They are typically obtained from rodents but can also be obtained from human biopsies. The main advantages of organotypic cultures are the preservation of the cytoarchitectonic complexity along with the circuit connections present in the tissue of origin, and the possibility of pursuing long-term studies as opposed to acute brain slices or conventional primary cell cultures [162].

This model may be considered to be bridging the gap between the in vitro 2D culture and the in vivo worlds. Its relevance is particularly evident when the organotypic culture is established from human biopsy samples, as it enables personalized medicine studies in long-lasting biological substrates obtained directly from the patient.

Recently, the concept of organotypic long-term culture has been applied to brain organoids. This strategy has been shown to ameliorate network vitality and electrical activity, thanks to the improved tissue oxygenation [126,141,159].

### 3.2. Combined MEMS and Microfluidics Based Platforms to Reproduce or Control Dynamic Extrinsic Microenvironments

From development to adulthood, tissues and organs are continuously exposed to spatial and temporal gradients of signaling molecules, distribution of oxygen and nutrients. Fluid flows (e.g., interstitial, blood, lymph) ensure their delivery throughout the body and also provide for the expulsion of waste products from it. Therefore, in the effort to generate in vitro systems that reproduce the physiology of tissues and organs, a substantial aspect to consider is the extrinsic microenvironment and its dynamic nature. In this regard, despite the capacity to mimic the multicellular layered organization of the brain [163] as well as vasculature-like structures [164], bioengineered brain tissue cannot per se offer continuous media exchange, molecular gradients, or flow dynamics found in native brain tissue. As microfluidics provides tools for manipulating and precisely controlling fluids, its integration in culture systems is crucial to obtain both spatially and temporally finely regulated microenvironments [165]. To this end, combined MEMS and microfluidics enable unified platforms for BoC biotechnology, wherein some fundamental features of organ structure and physiology can be recreated. Not less important, these platforms enable high-throughput screening studies thanks to the highly reproducible culture conditions and drug concentrations, while upscaling the screening processes [155,166], thus paving the way to personalized medicine. Here, we describe the most relevant MEMS–microfluidics platforms for BoC biotechnology and their applications.

Vasculature—Despite the several attempts to induce angiogenesis in brain organoids, there is no established approach to achieve the formation of functional blood vessels. The most remarkable works have obtained vascularized brain organoids by means of two strategies: (i) grafting brain organoids in the rodent cortex to achieve a passive vascularization process, i.e., blood vessel of the host brain penetrating the organoid [138], and (ii) co-culturing human stem and umbilical vein endothelial cells, and subsequent grafting of the so-formed vascularized brain organoid into the rodent cortex [167]. However, these approaches lack the pumping mechanism of blood vessels, which is provided for by the heart in the intact organism. Microfluidics can be used to make up for the difficulties in obtaining fully vascularized bioengineered brain tissue, by enabling a continuous supply of media and nutrients and an efficient elimination of waste molecules [168,169,170,171].

**Figure 6:**
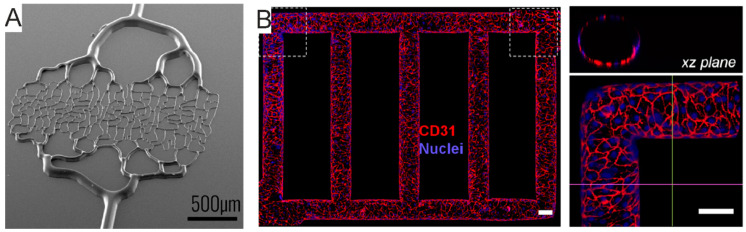
Microfluidics pseudo-vasculature. (**A**) Artificial vascular networks. Scanning electron microscopy image of the SU8 mold obtained by backside lithography. Adapted with permission from [169]. Copyright © The Royal Society of Chemistry 2019. (**B**) Endothlialized microfluidics pseudo-vessels. Z-stack projection of horizontal confocal sections of the overall network (left) and close-up views of the corners (right) indicated by the dashed boxes in the left panel. Scale bar: 100 μm. Adapted from [171].

Microfluidics-based pseudo-vasculature can be integrated in microscaffolds supporting 3D cell cultures [172], printed within hydrogel-based scaffolds [173], or by lithography techniques [169,171]. The latter has permitted the design of artificial vasculature faithfully replicating the geometries, size and distribution hierarchy of the native vasculature [169] (Figure 6A) or of endothelialized perfusion systems [171] (Figure 6B). In addition, the combination of MEMS with microfluidics has recently brought about a model of functional BBB-on-chip, which promises to be a powerful tool for screening brain-targeting drugs for their ability to pass the BBB under finely controlled conditions [13]. Note that BBB spheroids have also been recently described [117]. However, although they represent a valuable tool to study the BBB penetration by drugs, they do not offer the physical confinement of the different involved cellular actors. Such a feature may be required to dissect the contribution of different cell types to drug penetration and, presently, it is only possible to attain it with microfluidics platforms.

Biochemical gradients—Concentration gradients depend upon the geometry of the gradient forming region, the diffusive or convective transport of the molecules, and the porosity of the 3D matrix contained in the microchannels [174,175]. The generation of such gradients is so versatile that it can find numerous applications, from studying cell migration or morphogenesis, e.g., neurite pathfinding [176], differentiation of neural progenitor cells into neurons [177], to replicating complex tissue and organ morphogenesis, reminiscent of embryonic developmental processes. In this regard, particularly noteworthy is the recently described neural tube-on-chip, obtained via application of simultaneous opposing and orthogonal gradients of growth factors [178].

Mechanical cues—Mechanical environmental cues like interstitial flow, flow-induced shear stress, and matrix stiffness directly affect cellular mechanobiology, i.e., the ability of the cells to sense mechanical stimuli and convert them in electrochemical and molecular processes [55,179,180]. As mechanical stimuli are particularly important in embryonic development as well as in tissue homeostasis, mechanical stress can lead to dysfunctional tissue/organ regulation. Microfluidic devices can integrate microenvironmental mechanical stimuli, and gradients thereof [175,181]. As such, they are catching attention as new tools to both investigate and control the mechanobiology of organ-on-chip. Indeed, they enable the study of the cell migration response to mechanotransduction of fluid stresses within cancer spheroids [180], neurite growth in response to ECM stiffness gradients [179], or the response of cultured neurons [182] as well as of model organisms [183] to mechanical stimuli delivered by pneumatic actuators. In the context of BoC, the implementation of mechanical factors may enable fine-tuning of organoid development and maturation, for which mechanobiology has so far been overlooked, whereas the primary focus in the field is the design and optimization of differentiation and growth factors cocktails.

**Figure 7:**
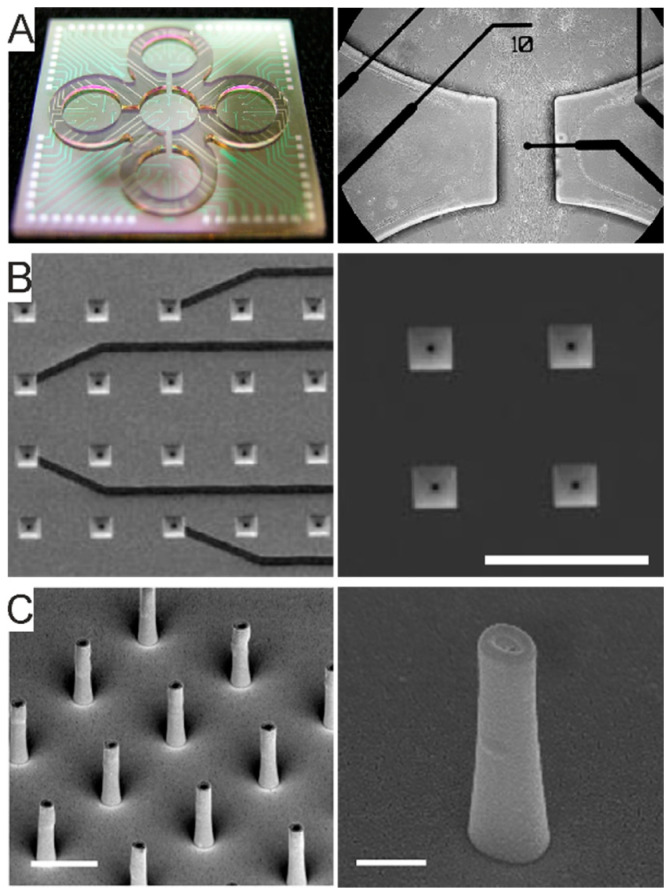
Microfluidics MEA devices for compartmentalized BoC electrophysiology. (**A**) MEA for compartmentalized neuronal networks based on physical confinement. Left—MEA with EPON SU-8 clustering structures for investigating interconnected neuronal populations. Adapted with permission from [195]. Copyright © 2014, Springer Science Business Media New York. Right—close-up view of an interconnecting channel within the MEA shown on the left, accommodating a cultured hippocampal network. Copyright © 2014 Elsevier B.V. (**B**) Micro-sieve MEA. Left—poly-silicon patterned electrode layer consisting of contact electrodes, lead wires and sieving structures accommodating the sensing electrodes in their pyramidal shaped pores. Right—scanning electron microscopy images of the top side of the silicon sieving structure. Pore base length: 20 μm. Pore distribution pitch: 70 μm. Scale bar: 50 μm. Adapted from [193]. (**C**). Microfluidic MEA. Left—scanning electron microscopy image of the hollow nanostructures fabricated on planar MEA electrodes. Scale bar: 2 μm. Right—magnification of a hollow nanostructure. Scale bar: 500 nm. From [194].

Compartmentalization—Combined MEMS and microfluidics enable network and cellular studies that would not otherwise be possible with conventional culturing techniques. In fact, microfabrication makes it possible to create compartmentalized culturing architectures through physical confinement achieved with the use of microstructures. Compartmentalization can be deployed at different scales, from neuronal networks (e.g., region-specific organotypic brain slices or cultured neuronal clusters), to the single neuron, down to different portions of a single neuron (i.e., soma, axon, dendrite, synapse) [28]. Compartmentalized neuronal networks are fundamental to study the communication between different brain regions, and between the local circuits present within the same brain region, including elucidating the mechanisms of connection directionality. These platforms have been used to study the development of functional connections and the resulting electrical synchronization in combined cortex–hippocampus organotypic slices using conventional field potential recording [184], or to address inter-regional connections in multi-regional primary neuronal cultures [51] and modularity-driven electrical patterns in primary cortical neurons coupled to MEAs [38] (Figure 7A). Along with the spatial confinement, these platforms make it possible to pursue chronic, spatially-restricted manipulations of pre- and post-synaptic neuronal circuits (e.g., by pharmacological or electrical modulation), or of biochemical and mechanical cues. As such, not only are they highly valuable to better understand the mechanisms of brain function and dysfunction at the network level, they also enable the operation of a fine control over the microenvironment factors involved in brain development, which are highly relevant to advance the brain tissue bioengineering field.

Single neuron compartmentalization is key to addressing cellular physiological functions such as axonal transport, synapse-to-nucleus signaling (via separated pre- and post-synaptic compartments, as in [185]), biochemical and electrochemical gradients across the neuronal tree axis, as well as the cellular pathophysiology of brain disorders. In this regard, microfluidics-compartmentalized neuron and astrocytes cultures can be used, e.g., to study neuroinflammation via biochemical analysis of cell–cell interactions [186]; compartmentalized neuronal segments can be deployed to study the mechanisms of axonal regeneration upon injury [187], the mechanical gradient-dependent neurite growth [179], axon myelination [188], and the role of the neurovascular unit in neurodegenerative disorders (e.g., motor neuron degeneration) [189]. These devices can be used for 2D cultures (as in [179,186,187]) or with (3D) neurospheroids (as in [189]). Nonetheless, as opposed to devices for network compartmentalization, they do not incorporate microelectrodes for electrophysiology measurements. In this regard, recent advancements in microfabrication techniques have brought micro-sieve array devices wherein the micro-sieves are scaled to the neuron size, enabling hydrodynamic single-cell capture [190,191,192] (Figure 7B). These devices have also been demonstrated to be suitable for integration of microelectrodes for electrophysiological measurements with single neuron resolution [193]. More recently, microfluidic MEAs have been developed, which allow simultaneous electrical recording and localized drug delivery [194] (Figure 7C), thanks to which it is possible to address the effect of biochemical modulation of small neuronal ensembles on the overall network activity.

## 4. Brain-on-Chip Electrophysiology: Fabrication, Features and Applications of Established and Emerging Tools

The measure of neuronal electrical signals requires that an electrode is located in the close proximity of neuronal cells, along with different hardware components that allow amplification, filtering and sampling of electrode potential changes with respect to a stable pseudo-reference electrode. Nowadays, the plethora of available electrophysiology techniques enable addressing several different electrical features of excitable tissues. These features range from the biophysical properties of single neurons (e.g., patch-clamp recording), to the concerted activity of small neuronal ensembles (extracellular multi-unit activity recording), and to the activity of extended neuronal networks (extracellular local field potential recording). The *conventional* electrophysiology techniques include those based on the use of glass micropipettes for patch-clamp and field potential recording, and tungsten electrode wires for single- or multi-unit recording. More recently, MEMS technologies have enabled the design and fabrication of recording/stimulation devices that have surpassed the technical limitations of canonical glass pipettes or wire electrodes [196]. In fact, in addition to improving the electrode fabrication reproducibility of these hand-made devices, the density of recording sites can be significantly increased, thus enabling simultaneous sub-millisecond recording of electrical signals from several locations with a greater spatial resolution than conventional techniques, which in turn permits the resolution of electrical activity from the network level down to the single neuron. Notably, devices fabricated on polymeric substrates permit significant reduction in the mechanical stress on the tissue. Finally, and most importantly, Finally, and importantly, MEMS-based electrode arrays carry the unique potential of being fully incorporated within BoC platforms for long-term electrophysiological studies, as opposed to *conventional* electrophysiology tools.

In this section, we therefore focus on electrode arrays, with the aim of distinguishing them from the application standpoint, and, in particular, between devices for surface and depth electrophysiology recording. Below, we first provide some introductory considerations about their fabrication. In this respect, several factors need to be taken into account for their design, as they determine the quality of the recorded electrical signals in terms of signal-to-noise ratio (SNR), the spatial resolution that can be possibly achieved (e.g., single unit or multi-unit), and the device stability in terms of practically feasible recording duration (acute or short-term versus chronic or long-term).

First, the electrode material, its surface properties and its shape determine how tightly the electrode couples to the cell(s) or to the tissue. The combination of these two factors not only contributes to the quality of the recorded electrical signal, but also to the electrode biocompatibility and thereby how long it can efficiently interface with the biological sample.

Second, as the electrode density within an array dictates the spatial resolution by which a network of neurons is resolved, the electrode array layout must be adapted to the experimental paradigm. In order to increase the electrode density, a highly challenging aspect is the routing scheme of each electrode to a dedicated read-out converter. Typically, the increase in the electrode density comes at the expense of the variety of the electrode array layout and of the possible integration of 2D or 3D electrode features. The latter is another fundamental interfacing property of electrode devices. This property must be suitably adapted with respect to the probed biological samples, including in vitro 2D cell cultures and 3D brain tissue models such as organoids, or in vivo deep brain structures or superficial cortical layers.

Lastly, electrode arrays can be augmented with additional functionalities, such as microfluidics or other sensors for physical or chemical parameters. These functionalities can be used to shape neural networks, to add a chemical read-out device or to supply a more suitable extrinsic environment to the biological specimen.

All these aspects need to be taken into account for the design and fabrication of BoC devices. Their optimization requires an important inter-disciplinary research effort, involving materials science, electronic engineering, microfabrication and microfluidics, and their convergence with neurobiology.

### 4.1. Fabrication

#### 4.1.1. Inorganic and Organic Electrode Materials

Materials strongly influence the performance of microelectrodes in terms of SNR, signal distortion effects [197], and charge capacity [198]. Therefore, the choice of materials for the fabrication of MEAs strongly depends on the target application performances for single unit versus local field potential recording and electrical stimulation. Here, we provide a primer of inorganic and organic materials that are most commonly used for MEAs. For an in-depth technical description and materials characteristics, the reader can refer to specific reviews [199,200,201].

Inorganic materials are the most commonly used in MEAs. Among them, noble metals like gold or platinum are biocompatible and low impedance materials, and, as such, they are theoretically ideal for electrophysiology applications. However, when it comes to microelectrodes, achieving a low electrode impedance using such plain metal conductors is challenging. This is because the electrode impedance increases as the electrode diameter decreases, thus affecting the electrode noise performances. Moreover, when microelectrodes are coupled to cells or tissue, the cleft between electrode and biological specimen gives rise to the so-called sealing resistance, which can be in the MΩ range, and thus massively affects the amplitude of the recorded signal [202]. To overcome these issues, an additional layer of a porous inorganic material such as titanium nitride (TiN) [203] or platinum black (PtB) [204] can be deposited on top of the metal conductor. This increases the actual electrode area while decreasing the sealing resistance [205] as required to improve the SNR in electrophysiology recording. Nonetheless, one major drawback of these electrodes is their opacity, which makes simultaneous optical imaging difficult. Recent advances in materials have brought optically transparent electrodes, such as TiN-coated electrodes [206].

Organic materials have more recently emerged as a strategy to coat microelectrodes. This is because of their wide range of chemical structures, which offers more surface functionalization possibilities, such as incorporating cell-adhesion molecules, growth factors [207], entrapping and releasing drugs [208], or making use of bioactive molecules [209,210]. Moreover, organic coatings are softer than metallic electrodes. Thus, their stiffness can more closely resemble that of brain tissue [211] to reduce the electrode–tissue mechanical mismatch and thereby the risk of inflammatory responses [212,213]. Lastly, the electrode surface roughness greatly increases its charge injection limits [214,215], enabling safer electrical stimulation at high intensities while decreasing the risk of eliciting irreversible tissue damage consequent to faradaic reactions [216]. Most commonly, organic electrodes rely on carbon nanotube coating, which can lower the impedance of the metal component of the electrode [217], and conductive polymers such as polypyrrole [209,218] or various modifications of poly(3,4-ethylenedioxythiophene) polystyrene sulfonate (PEDOT:PSS) [219]. These materials are extensively used as coating for electrodes in neuroprosthetics devices [218,220,221,222]. Other than coatings, organic semiconductors have been also successfully employed as electrodes for action potential recordings as well as for detecting neurotransmitters at different neuronal network levels [223,224,225]. A promising avenue to improve the biomimetic properties of organic electrode coatings is to combine conducting polymers such as PEDOT with hydrogels to further decrease their stiffness to about 1 MPa, which is within the same order of magnitude as the stiffness of brain tissue [226].

#### 4.1.2. Passive and Active Devices

Advances in microfabrication processes and microelectronics have been key drivers for the development of the first MEAs in the 1970s. As reviewed by J. Pine [227], one of the pioneers in MEA technology, electrode arrays for in vitro electro-physiology were initially demonstrated by C. Thomas [204], G. Gross [228] and J. Pine [229] in the 1970s. Over the last 40 years, the versatility of micro- and nano-fabrication processes has allowed to bring forward these first prototypes to commercial and widely used products. Further, advances in micro-/nano-structuring technology have opened-up new capabilities to optimize the electrode–electrolyte–neuron interface [230], by applying different electrode materials (e.g., Au, Pt and PtB, IrOx, TiN, or carbon-based materials) [198,231,232] and by addressing the interfacing properties of planar or 3D electrode morphologies [233,234,235,236]. Other studies have addressed the challenge of increasing the number and density of electrodes [237] as well as the integration of additional lab-on-chip functionalities, such as microfluidics for controlled compound delivery, or physico-chemical sensors for environmental parameters.

Nowadays, MEAs can be distinguished into two major generations of passive and active devices [238], based on the used fabrication technology and off-chip versus on-chip signal conditioning (e.g., filtering, amplification, analog-to-digital conversion). In passive MEAs, signal conditioning is performed off-chip by individually routing each microelectrode to a read-out pin, which is wired to an external amplifier. In active MEAs, signal conditioning and multiplexing is performed on-chip by means of integrated electronic circuits.

**Figure 8:**
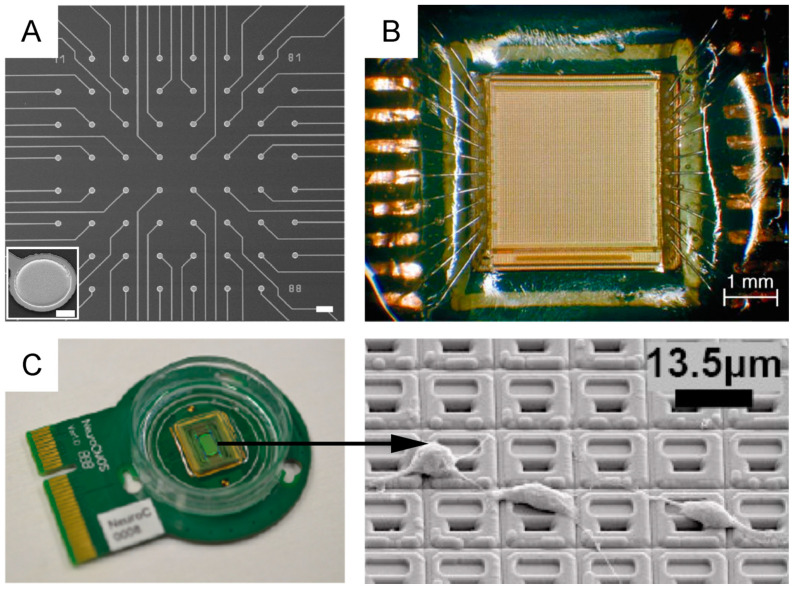
Active and passive planar MEAs. (**A**) Passive planar MEA (scale bar: 100 μm). The inset shows the scanning electron microscopy image of a TiN microelectrode (scale bar: 10 μm. Adapted with permission from [203]. Copyright © 1998 Elsevier Science B.V. (**B**) Active pixel sensor MEA made of 4096 gold microelectrodes. From [246] with permission. Copyright © 2004 Elsevier B.V. (**C**) High-density (HD)-MEA with ~60000 electrodes. Left—biocompatible chip packaging and PCB. Right—scanning electron microscopy image of the chip surface, showing in-house post-processed Pt-electrodes and dissociated primary rat cortical neurons, cultured on top. Adapted with permission from [249].

Passive MEAs (Figure 8A) represent the first and mostly used generation of these devices. They are typically made of planar electrodes of 10–30 µm in diameter, electrode separations of 100–500 µm and array sizes of 60, 128 or 256 electrodes organized in a regular square or other geometric configurations (*cf.* §4.2.1). Among passive MEAs, it is also worth mentioning 3D MEAs, developed since the past two decades [239], which are, however, somewhat less popular than planar MEAs.

Passive MEAs are obtained with micro-/nano-structuring processes and are characterized by individually wired on-chip microelectrode sites. Several fabrication processes have been proposed in the literature due to differences in the target substrate (silicon, glass or polymers), electrode material, size or morphology, and geometry (planar versus 3D). An example of a fabrication protocol for in vitro/ex vivo applications is reported in [240]. Briefly, the basic steps of the entire clean-room process for planar MEA fabrication consists of the following: (i) design and realization of two patterned masks to structure a metal layer and the apertures in the insulation layer; (ii) metal deposition and lift-off to structure the electrode sites, interconnecting leads and contact pads; (iii) deposition of the insulator layer; (iv) a second photolithography and opening of microelectrode sites and contact pads through the insulator; (v) cleaning, wafer dicing and chip mounting on an interconnecting printed circuit board (PCB) by wire-bonding. The mounted device is completed with a glass or plastic reservoir that provides a chamber to accommodate the biological sample along with the physiological medium to keep the sample alive. Fabrication of 3D MEAs may be attained primarily by two strategies using a similar approach as for planar electrodes. Protruding Pt electrodes can be electroplated atop of the active area of planar electrodes previously patterned using a lift-off process, and exposed by etching, as in [233] for 3D Pt hillocks. Alternatively, tip-shaped electrodes can be structured on 3D patterned substrates. Specifically, as in [234,239], 3D tip-shaped electrodes are obtained by deposition on a glass substrate that had previously been etched to form a 3D topology at the electrode sites. In [235,236] arrays of Si-tip electrodes were obtained by etching the silicon substrate at the electrode sites and by successively structuring a metal and insulating layer for the electrodes. Moreover, 3D MEAs have been also engineered with protruding nanostructures which can locally increase the cell-chip coupling and eventually and eventually achieve in-cell recording. These devices can be obtained by electron beam patterning or by metal deposition through focused ion beam [241,242].

The on-chip electrode-pad wiring of passive MEAs uses metal leads and imposes spatial constraints for the electrode array layout. In addition, off-chip connections to external amplifiers contribute to increasing the total noise level and crosstalk. In fact, such long leads (in the cm-range) inevitably pick-up parasitic capacitances, which not only contribute to decreasing the SNR, but can also account for signal distortion [197,243]. Furthermore, as the number of electrodes increases to several hundreds to thousands, it becomes harder, if not unfeasible, to route all electrodes to their respective pins, due to unavoidable leads intersect which, in turn, creates a short-circuit.

Active MEAs (Figure 8B,C) represent the second and more recent generation of these devices, which have brought a major breakthrough to overcome the limitations of passive MEA devices. The active MEA technology has been introduced twenty years ago and consists of monolithic complementary metal-oxide semiconductor (CMOS) circuits [237,243,244,245,246] (Figure 8B). The shift to CMOS technology, allowing the integration of on-chip circuit architectures, has enabled the fabrication of high-density (HD)-MEAs with on-chip signal conditioning circuits that avoid noise issues arising from long wiring. Using these devices, it is nowadays possible to record the spontaneous and electrically-evoked neural activity from several thousands of closely spaced (<20 µm) microelectrodes [243,247,248].

Within CMOS HD-MEAs, the current state-of-the-art consists of ~60 thousand electrodes on a 4.5 × 2.4 mm^2^ area, with a 5 × 5 μm^2^ electrode surface area and a 13 μm inter-electrode pitch [249] (Figure 8C). Together with sub-millisecond time resolution, these devices provide an unprecedented spatiotemporal resolution to access cellular and network level activity [250,251,252] down to the subcellular scale [248,253]. Further, the advent of CMOS-based HD-MEA technology has allowed the integration, within the same lab-on-chip platform, of multiple functionalities, including impedance mapping, thermal monitoring or neurotransmitter detection [249,254,255,256]. It needs to be mentioned, however, that, despite its outstanding performance and potential, CMOS-HD-MEA technology carries the major drawback of being based on Si substrates. This leads to stiff and non-transparent devices, which makes it harder to couple it with live imaging techniques.

The fabrication of active MEAs relies on CMOS foundries that realize the microelectronic circuit design. So far, proposed devices were based on the 0.13, 0.18 or 0.35 µm CMOS technology nodes. Microelectronic fabrication is followed by post-processing techniques that are used to modify the electrode morphology and materials. This post-processing is a required step because CMOS fabrication processes typically rely on Al-alloy metal layers and do not include adequate electrode materials.

**Figure 9:**
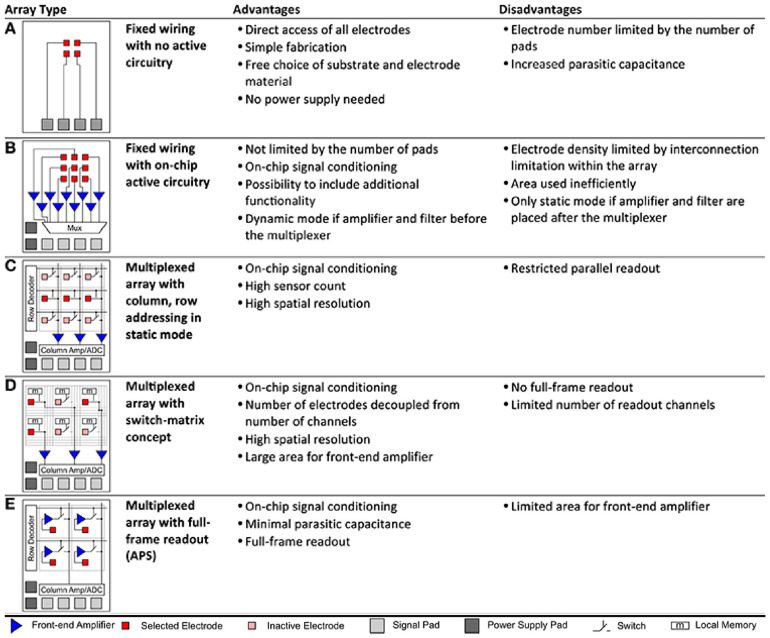
Passive and active MEA architectures. Overview of the different types of MEA architectures, their advantages and limitations. A and B. In fixed wiring architectures, the electrodes are directly connected either (**A**) to signal pads with no active circuitry or (**B**) directly connected to on-chip active circuitry for signal conditioning. C–E. Multiplexed arrays. Signals can be multiplexed to the signal pads by (**C**) static (column, row) addressing, (**D**) a switch-matrix, which adds more routing resources within the array for more flexible addressing, or (**E**) integrating the front-end circuits underneath each electrode in active pixels sensor (APS) MEAs for fast-speed, full-frame readout. Adapted from [238].

As recently reviewed by [238], passive MEAs are limited by fixed wiring, whereas active MEAs enable the added feature of array multiplexing (Figure 9), either by integrating a switch-matrix or using the active pixels sensor (APS) concept, or a combination of the two [257]. The switch-matrix enables the integration of a very dense electrode array and the simultaneous read-out from a subset of electrodes, as defined by the number of front-end circuits integrated on the sides of the active area. The APS integrates the front-end circuits underneath each electrode, enabling whole-array read-out.

### 4.2. Surface Electrophysiology

#### Planar Microelectrode Arrays

**Figure 10:**
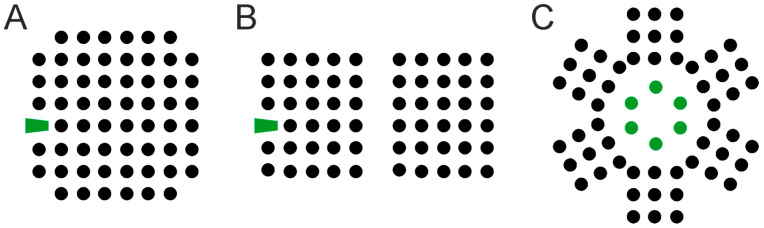
MEA layouts commonly available for 60-channels planar MEA. (**A**) 8 × 8 layout. (**B**) Two 5 × 6 quadrants layout. (**C**) Multi-well layout consisting of 6 wells accommodating a 3 × 3 electrode grid each. The green dots or trapezoids are the reference electrodes.

Planar MEAs are the current gold standard for multi-site surface electrophysiology recording. Typically, the microelectrodes lie on a flat substrate (usually glass or silicon) and are arranged in different layouts, most commonly a square grid, depending on the application purpose for which they are designed. The most common layout for 60-electrodes MEAs is a single 8 × 8 grid (Figure 10A). This layout is typically used for general purpose applications, like cell culture or hippocampal acute or organotypic slice recording. The 6 × 10 grid is less frequently used, but it is more useful in the case of large biological samples, which may expand vertically beyond the typical square area of an 8 × 8 MEA. Other layouts are available for special applications, such as the quadrants and the multi-well MEA. The quadrant type consists of two or more electrode quadrants within the same recording chamber (Figure 10B). Since the different quadrants are not physically confined, this MEA layout is particularly useful for modular cell culture studies, where the modules are connected through micro-channels achieved with the use of PDMS masks. The multi-well type (Figure 10C) enables the simultaneous monitoring of several microcultures and is particularly helpful to more rapidly address physiological and pathological network phenomena, as well as for pharmacology and electroceutical studies.

Among planar MEAs, a particular mention should be made of perforated MEA (pMEA). These devices combine microfluidics with surface recordings and were developed specifically for acute brain slice applications requiring improved oxygen supply to the tissue, which is known to be impaired by the tissue adherence to the non-porous MEA substrate. As this is commonly an issue with any 3D biological sample coupled to a MEA, the pMEA may be a useful electrophysiology read-out tool for BoC. However, to the best of our knowledge, no one has so far made use of these devices for BoC electrophysiology.

Surface electrophysiology recording based on planar MEA is a practical and quite easy technique to implement. Planar MEAs have indeed been successfully used to record the electrical activity generated by neural spheroids, intact brain organoids and organoid slices (*cf.* §3.1.2), as well as by human iPSC-derived neuronal networks [258]. However, this is not yet the mainstream technique to address the functional features of bioengineered brain tissue. Moreover, planar MEA electrophysiology carries the limitation of enabling the detection of neuronal signals from the first few tens of microns depth from the electrode–tissue contact. Therefore, in the case of acute or organotypic brain slices, thick 3D cell cultures, neural spheroids or brain organoids, the electrical signals generated by cells populating the deeper layers of the sample remain inaccessible. Overall, this contributes to the loss of useful information about the function of 3D biological samples. This aspect becomes particularly relevant in the case of BoC electrophysiology, where characterizing the sample electrical features in the three dimensions is an asset for developmental functional studies as well as for other studies, such as investigation of the structure–function relationship of the brain tissue replica.

### 4.3. Depth Electrophysiology

Electrical access to the inner layers of a 3D biological sample may be attained by the use of three main strategies: (i) protruding electrodes, wherein the specimen is laid on the array and is penetrated from the bottom, (ii) silicon array probes, which are inserted in the specimen at the desired location, or (iii) incorporated electronics, which become an integral part of the specimen, whether they are injected in it or naturally incorporated by the 3D structure during its formation.

#### 4.3.1. Protruding Structure Array

Since the late 1990’s there has been much research dedicated to the development of protruding electrodes that can access the electrical signals generated from deep within 3D biological samples, while improving the cell/tissue-electrode coupling. The latter depends on the actual electrode area contacting the sample [259], for which planar electrodes have started to be augmented with a variety of micro- and nano-structured topographies [260,261].

**Figure 11:**
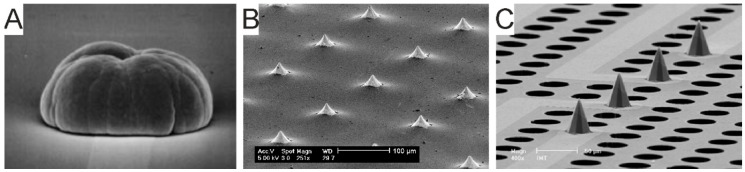
Protruding microstructures. (**A**) Electroplated Pt hillock. Adapted with permission from [233]. (**B**) Tip-shaped glass microelectrodes obtained by etching the glass substrate and by structuring the electrode metal and EPON SU-8 insulator. Adapted with permission from [239]. (**C**) Si-tip electrodes on a porous MEA obtained by etching the silicon substrate before structuring the electrode materials. Adapted with permission from [195]. Copyright © 2014, Springer Science Business Media New York.

Protruding electrode microstructures were first described in the late 1990s [233], where the 3D structures were electroplated Pt hillocks (Figure 11A). Modern 3D MEAs typically consist of tip-shaped microelectrodes [195,235,239] (Figure 11B,C). The 3D tip-shaped electrodes can vary in base area and height and can penetrate 40–100 µm deep into the biological specimen. More recently, Colistra and colleagues have described gold ball-shaped 3D electrodes [262].

**Figure 12:**
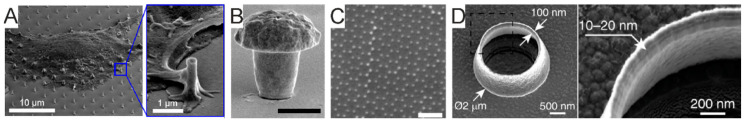
Protruding nanostructures. (**A**) Nanopillars. Scanning electron microscopy image of an HL-1 cell on a quartz substrate with nanopillars. The inset shows the magnification of a nanopillars and the preservation of the membrane protrusions in contract with it. From [271]. (**B**) Mushroom stalks. Scanning electron microscopy images of a gold mushroom-shaped protrusion. Cap diameter: 1.5 μm. Scale bar: 1 μm. Adapted from [266]. (**C**) Nanospheres. Scanning electron microscopy image of a Pt-nanostructured microelectrode with a 160 nm thick Ag layer deposition over 456 nm polystyrene beads. Scale bar: 1 μm. Adapted with permission from [269]. Copyright © 2013 Elsevier B.V. (**D**) Nanovolcanoes. Left—scanning electron microscopy image of an Au nano-patterned nanovolcano. Right—magnification of the nanowall rim indicated by the dashed black box. Adapted from [270].

Protruding nanostructures include a plethora of geometries, such as nanopillars [263,264] (Figure 12A), mushroom stalks [265,266,267,268] (Figure 12B), nanospheres [269] (Figure 12C), and so-called nanovolcanoes [270] (Figure 12D). The local curvatures of these structures seem to match the native curvature detection of the cell membrane, which spontaneously wraps around these structures ensuing an endocytosis process [271]. In turn, this not only leads to a narrow gap between electrode and cell, it also increases the overall coverage of the electrode surface by the cell membrane, because of the 3D spatial rearrangement of the latter.

Overall, the so-improved electrode–cell coupling leads to an enhanced SNR [272], and to a significant reduction in the mechanical perturbations to the natural extracellular environment. In fact, protruding nanostructures seem to increase electrode biocompatibility and cell viability in the long-term [273]. Lastly, protruding structures characterized by a low-curvature edge, such as nanopillars and nanovolcanoes, make it possible to reversibly electroporate the cell membrane to gain intracellular electrical access [274]. In the case of nanovolcanoes, it has been observed that the membrane may also porate spontaneously without any external stimulus [275]. This may lead to a new paradigm in signal detection by enabling access to high-SNR iuxtacellular signals when gathering single unit activity is the objective.

#### 4.3.2. Silicon Probes

**Figure 13:**
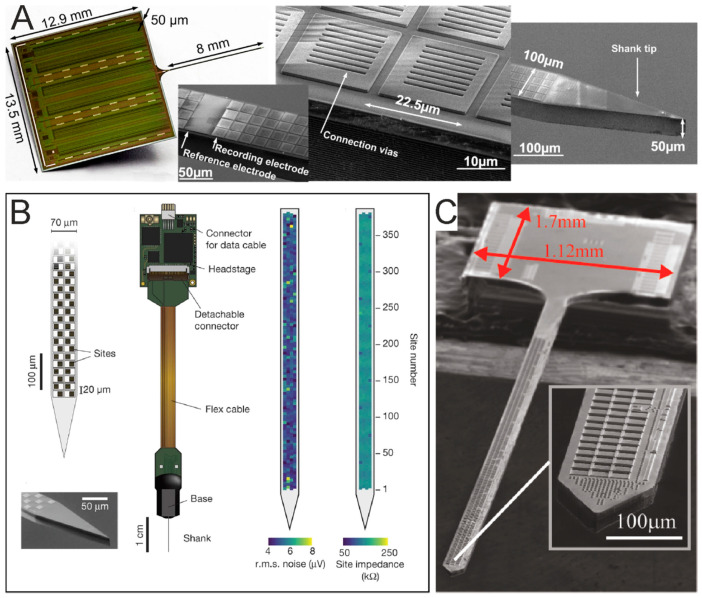
Silicon Probes. (**A**) Neuroseeker multiplexed probe with 1356 recording sites. From [278]. (**B**) Neuropixels probe with 384 simultaneous recording sites selectable from 960 electrodes. From [276] with permission. Copyright © 2017, Macmillan Publishers Limited, part of Springer Nature. (**C**) SiNAPS probe providing whole-array recording with 512 sites. Adapted from [281].

Silicon probes are micro-machined electrode arrays, which can be active or passive (*cf.* §4.1.2, reviewed in [196]), and consist of single or multiple implantable shanks populated by several planar electrodes (Figure 13). Designed to maximize the number of recording sites while minimizing the insertion-induced damage to the brain tissue [276,277,278,279], they are primarily conceived for in vivo use. However, they have recently also found application for brain organoid electrophysiology [142,159,280].

Silicon probes carry several advantages over protruding structure arrays. First, they can be inserted deep within the tissue at the desired location, as opposed to the poorly controllable tissue penetration site(s) attained with protruding structure arrays. Second, they can be removed and re-inserted with minimum damage to the tissue, allowing to probe several locations within the same sample, e.g., to search for active areas within the sample. Third, their design in terms of electrodes positioning, enables the simultaneous collection of signals from several distinct recording sites with a single probe. However, as the probe extends externally to the chamber compartment accommodating the organoid, this technique does not permit the preservation of sterile conditions and, as such, it only enables acute recordings, whereas time-course functional analyses within the same sample are not possible.

Silicon probes are most frequently fabricated by photolithographic patterning of thin films of conductors and insulators on a silicon substrate. This fabrication process permits fine-tuning of the probe geometrical specifications, i.e., shape and size, as well as the size, number and arrangement of the electrodes, or enhancement of the probe’s long-term performance with conductive polymer coatings, such as PEDOT [282]. MEMS technology allows tailoring these parameters to the specific application purpose, such as the location, density and architecture of the recorded local circuit. Nonetheless, the number of electrodes that can be accommodated within a single probe is still limited as compared, e.g., to their CMOS-MEA counterparts. The main constraint stems in the width of the embedded conductive leads (interconnects) required to route each electrode to the electronics of the external acquisition system. Previous work performed in awake rodents has shown that in a probe as thin as 20 µm, a shank wider than 60 µm implies a significant decline in the signal quality, possibly due to mechanical compression and damage to the neurons surrounding the micro-probe [283]. Considering the average size of bioengineered brain tissue as compared to the native mammalian counterpart, substantial advancements are needed to improve the compliance of silicon probes for BoC electrophysiology.

Along the roadmap towards the development of next-generation silicon probes, outstanding advances in MEMS technology have recently enabled, on the one end, smaller probe feature size along with a higher electrode density, and, on the other hand, the integration of active electronic components to provide electronic switching within the probe shanks, thereby decreasing the number of interconnects, and, consequently, the required shank size [276,278,281,284,285]. Alternative approaches to MEMS have also been recently proposed to fabricate HD-probes. These include electron-beam lithography, which has allowed the fabrication of a 64-channel probe with on-chip multiplexers [286], and integrated composite electrodes [287,288]. Among the latter, it is worth mentioning the microprobe based on a carbon-fiber core of very small (<7 μm) diameter, where poly(*p*-xylylene) coating was used as the insulation layer, and the recording site was functionalized via PEDOT or PEDOT:PSS coating [288]. This probe has been shown to exhibit a good mechanical compliance with brain tissue and to reduce inflammatory responses when tested in vivo.

#### 4.3.3. Tissue-Incorporated Electrodes

Tissue incorporated electrodes propose the concept of generating bio-artificial hybrids to achieve chronic in-tissue electrophysiological interfacing. Currently, two types of tissue-incorporated electrodes have been described as the latest development for BoC electrophysiology: (i) mesh electronics and (ii) untethered micro-devices.

**Figure 14:**
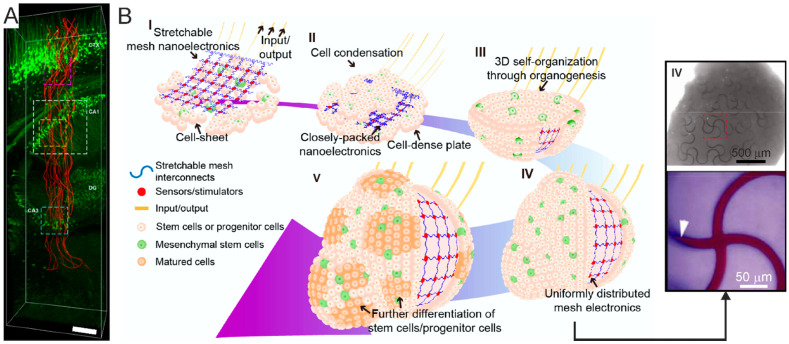
Mesh electronics for tissue-wide electrophysiology. (**A**) Injectable mesh electronics. 3D reconstruction of the interface between neurons (green) and the injected electrodes mesh (red) at 6 weeks post-implantation. The mesh spans the cortex and reaches the hippocampus. Scale bar, 200 μm. CA1: *Cornu Ammonis* 1. CA3: *Cornu Ammonis* 3. DG: Dentate Gyrus. Adapted with permission from [291]. (**B**) Left—schematic rendition of the stepwise assembly (I-IV) of the mesh electronics into the organoid through organogenesis. Right—bright-field image of a fully assembled organoid incorporating unfolded mesh electronics (step IV in the schematic rendition) and zoomed-in view of the region marked by the dashed red square. Adapted with permission from [143]. Copyright © 2019 American Chemical Society.

Mesh electronics (Figure 14) aim to fuse the concepts of array probes and flexible electronics in the effort to further reduce the footprint of the array, while minimizing the electrode–tissue mechanical mismatch [143,289]. These devices remove the substrate support from the electrodes and the electrode leads, and weave as little polymeric material as possible to create a device similar to a fishnet. Because they are soft and deformable, they can be injected into the tissue by means of a syringe [290,291] (Figure 14A). Alternatively, they can be incorporated within the bioengineered tissue during its formation, which could also be relevant to monitoring its developmental electrical features [143] (Figure 14B). To improve spontaneous integration and tight electrode–neuron binding, some mesh-electrode designs have proposed to integrate the active electrode array and the electric leads inside the mesh are scaled and morphologically similar to neurons and neurites [291]. This design has proven to further reduce the presence of inflammatory markers and to promote the formation of dense synaptic connections around the electrodes, thus enhancing the long-term integrability and signal quality of such devices.

Mesh electronics have overall proven to minimize inflammation and improve long-term biocompatibility, thanks to the very small footprint, which does not cause significant mechanical perturbations to the neural tissue. However, available recording meshes are typically hard-wired to an external system for signal conditioning and acquisition. Further development of this technology is required to provide robust electrical interconnects and enable wireless recording that would, in turn, permit the preservation of sterile conditions for long-term time-course functional studies.

**Figure 15:**
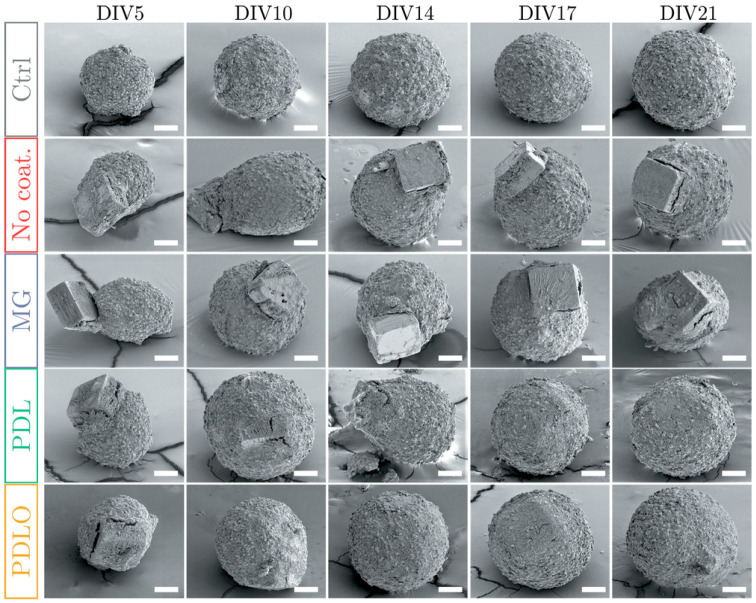
Functionalization-driven self-standing micro-device integration in 3D neural cultures. Scanning electron microscopy images of fixed neurospheroids taken at different days in vitro (DIV), showing the incorporation time course of functionalized and non-functionalized self-standing micro-devices. Ctrl: control condition, neurospheroid without microchip. No coating: non-functionalized micro-device. MG: matrigel. PDL: Poly-D-lysine. PDLO: poly-DL-ornithine. When the microchip is either non-coated or coated with MG, it remains at the periphery of the spheroid, whereas it is progressively incorporated within the spheroid when coated with either PDL or PDLO. Scale bar: 50 µm. From [302].

Untethered microscale biosensing devices can also be incorporated in forming 3D brain tissue models. Differently than mesh electronics, these micro-scale devices target the integration of untethered read-out capabilities with self-standing device systems that can be integrated extracellularly or even intracellularly. These integrated micro-devices can be interfaced using optical or radio frequency (RF) approaches, as well as ultrasounds, as under development for neural dusts that target stable chronic brain machine interfaces in vivo [292,293]. Optically interfaced self-standing microscopic silicon particle devices have been recently proposed for intracellular readouts [294,295] and demonstrated on isolated cells or 2D cell cultures for cell tracking using a barcode system [296], intracellular pressure sensing [297], or to implement multistage delivery systems [298]. Commercial radio frequency identification (RFID) chips of 460 × 480 µm^2^ in size integrated into re-aggregated iPSC-derived endoderm spheroids have been proposed to demonstrate phenotypic screenings of a pool of RFID-modified organoids [299]. However, these circuits do not yet provide the capability of electrophysiological signals read-out. To do so, a possible way consists in exploiting recent achievements in the massive downscaling of free-floating microelectronic independent biosensor nodes developed for CMOS-probes [281] to realize low-power active CMOS micro-devices of 10–100 µm in size [300,301]. Parallel to the micro-device circuit development, a recent work has demonstrated the possibility of using different surface functionalization to drive the localization of self-standing micro-devices into 3D neurospheroids (Figure 15) [302]. This work heralds the possibility of controlling the assembly of brain cells and micro-devices to achieve 3D brain tissue models with in-built untethered neuro-electronic interfacing functionalities.

### 4.4. Front-End Electronics

To operate MEMS-fabricated *passive* electrode arrays, including protruding electrodes, silicon probes and mesh-electrodes, the conventional approach consists in individually routing each electrode on-chip and in connecting them to a standalone instrument, providing signal conditioning and analog-to-digital conversion for each electrode channel. During the past decades, many different circuit solutions have been proposed to implement front-end amplifiers dedicated to retrieving low-frequency field potentials and weak action potential signals from extracellular microelectrodes. For many years, noise performances have been the major if not the only constraint in the design and implementation of such amplifiers, resulting in bulky solutions capable of recording from few electrodes that had to be installed in dedicated instrumentation racks [303]. Thanks to the advances in CMOS electronics that have occurred over the past 20 years, Application Specific Integrated Circuits (ASIC) dedicated to neural signal conditioning can now integrate hundreds to thousands of front-end amplifiers in small form-factor devices and offer novel opportunities for the integration of compact BoC platforms [304,305]. These microelectronic devices are also the result of innovative design approaches and novel circuit topologies that permit the improvement of opposing design constraints and trade-offs such as power consumption, noise performance and size of amplifiers. Many different research groups worldwide have proposed over the past few years highly integrated solutions that not only permit the retrieval of bio-electrical signals from extracellular microelectrodes, but also enable to implement on-chip subsequent signal processing steps (analog-to-digital conversion, spike detection, signal compression, wireless data transmission) toward the implementation of effective brain–machine interfaces (see e.g., [305,306,307,308,309]).

Despite major technological advances and the increasing capability in terms of data communication bandwidth and signal processing, wiring is the major scaling bottleneck of such an approach, caused by spatial limits of analog front-ends [310]. Typically, in fact, front-end amplifiers require a relatively large area to integrate a capacitive feedback architecture to achieve large AC gains and low-noise performances (both necessary to retrieve the small amplitude of extracellularly-recorded action potentials) while removing large DC offsets arising at the electrode–tissue interface [311]. Conversely, the DC-input architecture described in [312] for in vitro applications and in [281,313] for in vivo recordings were successfully demonstrated as a minimum area solution for highly-scalable neuroelectronic interfaces. In parallel, an important outcome of this research effort on front-end electronics for neural signals recording are low-power integrated circuit components that can be used in compact hybrid platforms to advance the interfacing of passive electrode arrays for a broad range of applications, including electroencephalography EEG recording systems [314,315] or BoC.

## 5. Tools to Complement Electrical Signals Read-Out

Electrical signals read-out can be complemented by a plethora of parameter-sensing tools to achieve a multi-parameter sensing BoC platform that can provide a more complete picture of the functional features of bioengineered brain tissue. These include, among others, oxygen and pH sensing, neurotransmitter release detection, calcium and voltage indicators combined with imaging techniques. Additionally, optogenetics can be used to probe and control the electrical activity of neurons as well as to guide cell fate and migration. Altogether, these tools permit an in-depth analysis of tissue function as well as a fine control over its structure throughout development and maturation. Here, we focus on the most deployed complementary tools, i.e., calcium and voltage imaging, and optogenetics.

### 5.1. Calcium Imaging

Calcium imaging is an optical approach used to monitor the calcium-mediated activity of cells, here specifically of neurons and non-neuronal cells within a bioengineered brain tissue. Like other imaging techniques, calcium imaging offers the advantages of non-invasiveness and specific cell-type targeting, along with high and flexible spatial resolution, wherein the activity of multiple cells can be distinguished yet simultaneously visualized [316]. As the electrical activity is always associated with a variation of calcium ion flow, calcium imaging is considered a complementary tool to electrophysiological readout, where pinpointing the simultaneous activation of single cells within a network is necessary, yet not feasible with other techniques.

Calcium imaging exploits fluorescent calcium biosensors, i.e., molecules that respond to calcium binding with changes in their fluorescence properties. These can be classified into two main categories according to their cell target specificity and time-constrained usage: (i) chemical (bath applied) calcium indicators and (ii) genetically-encoded calcium indicators (GECI).

Chemical biosensors (e.g., Fura, Bapta, Fluo4AM) are non-selective probes, which reveal the calcium activity of both neuronal and non-neuronal cells [317]. Therefore, although practical in their use via acute bath-application, they do not allow distinguishing the calcium signal generated by different neuronal populations, e.g., neurons and astrocytes. Such a distinction can only be made on a qualitative level, by looking at the calcium signal kinetics (for example, astrocytes are known to generate a slower signal than neurons). In addition, chemical calcium biosensors exhibit cytotoxicity, for which they can be used for acute investigations only.

GECI make use of a viral vector (e.g., adeno-associated-virus or lentivirus) to insert the fluorescent probe gene (e.g., gCAMP6) within the cell genome. The first GECI, Cameleon, was constructed by Roger Y. Tsien and coworkers in 1997, in order to detect and quantify the calcium levels in specific subcellular regions of HeLa cells [318]. This seminal work laid the foundation for the development of a plethora of GECI variants specific for neural cells [319]. GECI can be designed for selective expression in specific tissues and cell types, and, since they are directly encoded in the cell genome, they overcome the cytotoxicity limitation of chemical indicators, enabling the long-term monitoring of calcium activity [320,321]. In addition, genetically-encoded indicators carry the added advantage of multi-targeting, which can be achieved by the use of multiple viral vectors to co-deliver calcium indicators tagged with different fluorophores, each targeting a different cell type within the same biological sample. This allows the simultaneous monitoring of the activity of different cell types, e.g., neurons and astrocytes, and investigation of their functional interplay [322].

The combination of calcium biosensors with advanced volumetric (as opposed to area) functional imaging tools allows investigation of connectivity maps across the three dimensions of bioengineered brain tissue. This strategy opens new paths for interfacing large-scale 3D neuronal networks with imaging tools and novel 3D functional imaging approaches for screening and/or diagnostic purposes. Among advanced imaging technologies, it is worth mentioning (i) hybrid multiphoton microscopy and mesoscopy, (ii) wide-volume imaging, and (iii) volumetric Lissajous confocal microscopy at ultra-high scanning speed.

Hybrid multiphoton microscopy combines multiphoton laser scanning with a 3D scanning-line temporal-focusing system to provide functional and structural volumetric information, which allows detection of complex activity patterns of dense 3D neuronal networks of over one thousand cells at tens of voxels per second [323]. Hybrid two photon mesoscopy combines a two-photon fluorescence mesoscope endowed with a 5-mm diameter field of view and a Bessel focus scanning for volumetric imaging. This strategy has recently been demonstrated to resolve the dynamics of neural activity over the mesoscale with synaptic resolution in the awake mouse in vivo [324].

Wide-volume imaging combines wide field microscopy with a controllable phase modulation of the detection pathway. This approach enables monitoring of the activity of hundreds of neurons in a mm-size wide volume with single cell resolution [97].

Volumetric Lissajous confocal microscopy with ultra-high 3D scanning speed can achieve scanning rates in the kHz range [325]. With this approach, sample images are continuously acquired throughout a volume of tens of cubic microns and a post-processing step allows the selection of the most suitable volumetric imaging rate according to the desired spatial or temporal resolution.

Generally, along with the advanced imaging tools, accurate and possibly automated analysis tools are developed ad hoc to dissect structural and functional information of the networks and for deriving connectivity maps [326]. Moreover, advanced computational and statistical methods show promise to enable inferring the correlation of calcium signals with the underlying cellular and network dynamics from the biophysical standpoint [327,328,329]. These tools are expected to become a key driver for unraveling spatial and temporal information within large, millimeter-size, 3D samples using this easy and accessible imaging technique.

### 5.2. Voltage Imaging

Voltage imaging is an optical technique used to visualize neuron or neuronal network electrical activity via fluorescent probes that are sensitive to voltage changes. These probes, overall referred to as voltage indicators, are a class of molecules with relatively high temporal (sub-ms to 500 ms) and spatial (<50 μm) resolution, which are particularly suitable for mesoscopic scale neurophysiology studies, i.e., to address the spatiotemporal patterns of neuronal networks within a cortical column, or a specific brain area, as well as the interplay among connected areas comprised within a relatively large view field (1–2 cm^2^). As opposed to calcium biosensors, which represent the action potential output of neurons, voltage indicators provide information on the synaptic inputs impinging on the probed neuron, as they are sensitive to hyperpolarizing and subthreshold depolarizing membrane voltage fluctuations [330,331]. Thanks to these characteristics, voltage indicators enable all-optical electrophysiology approaches [332] which are not possible with the sole use of calcium indicators.

Two crucial parameters in voltage indicator design and application are (i) temporal response and (ii) sensitivity (or dynamic range). The latter is the steady-state fluorescence change (%) in response to a voltage step (mV) within a physiologically relevant range. Whereas the temporal response of voltage indicators determines their suitability to track fast action potentials, the sensitivity (%/mV) determines the SNR that can be achieved with this optical technique [333,334]. As explained below, some voltage indicators inherently exhibit slow responses and/or less sensitivity than others and, as such, the possibilities they can offer in terms of applications vary among and within different classes of biosensors.

Voltage indicators can be classified in voltage-sensitive dyes (VSD) and genetically-encoded voltage indicators (GEVI). VSD are water soluble amphiphilic molecules which localize nearby or intercalate within the cellular membrane and undergo photon emission as the membrane potential changes [335]. GEVI are engineered protein sensors built with different strategies (see below), which, similarly to genetically-encoded calcium indicators, are inserted into the cell genome with the use of vectors [334,336,337,338]. Whereas VSD are not cell-type-specific indicators, GEVI can leverage a plethora of promoters to target specific cellular phenotypes, allowing to selectively pinpoint the contribution of specific cell sub-population(s) to neuronal network dynamics.

VSD can be distinguished into two main categories based on the time resolution of their fluorescence emission: (i) slow-response VSD, which show a 1–500 milliseconds-range resolution; (ii) fast-response VSD, which show a sub-millisecond resolution [339]. Slow-response VSD are most commonly small molecules that undergo a voltage-dependent accumulation in the cell membrane and exhibit solvatochromic (solvent-dependent) fluorescence upon association with the lipid bilayer, e.g., merocyanine 540. Fast-response VSD, such as 4-ANEPPS [340,341] and ANNINE-6 [333] interact directly with the electric field across the cell membrane and their fluorescence shifts result from small wavelength changes that depend on the fluorophore dipole orientation relative to the membrane electric field. Whereas slow-response VSD typically present a very high sensitivity (80–100%/100 mV) [339,342] and, hence, a high SNR, fast-response VSD offer a lower sensitivity (2–50%/100 mV) [333], for which they require improvements to achieve a better SNR. In particular, the ANNINE VSD class offer an increased SNR thanks to their linear response to membrane potential changes and the faster temporal resolution as compared to other fast-response VSD. In addition, the ANNINE are particularly suitable for two-photon imaging and their key strengths are their high sensitivity (50%/100 mV), and negligible photobleaching and phototoxicity [333,339].

GEVI comprise three classes, according to their design scaffold, which also reflect their generation: class I—isolated voltage-sensitive domain, class II—opsin-based, and class III—chemigenetic or hybrid [338]. Isolated voltage-sensitive domain GEVI exploit the voltage-sensitive domain of ion channels, such as the K+ channel Kv3.1, whereas opsin-based GEVI exploit the voltage-dependent fluorescence of opsins [338,343,344]. Chemigenetic or hybrid GEVI can be regarded as a peculiar class of molecules, as they combine chemical VSD with genetically-encoded membrane proteins. The first GEVI, Flash, was introduced by Siegel and Isacoff in 1997 [345], around the same time of the description of the first genetically-encoded calcium indicator, Cameleon [318].

Isolated voltage-sensitive domain GEVI can be either Förster resonance energy transfer (FRET)-based, like the first-generation GEVI [346], or monochromatic indicators. This early generation of GEVI has enabled a direct voltage response, although at a slower time resolution than fast-response VSD. As such, they are reliable reporters of slow membrane potential transients, but they cannot resolve fast membrane potential excursions such as action potentials [336,343]. Opsin-based GEVI are a later class of GEVI, which provide a faster time resolution, thereby making it possible to resolve action potentials and study neuronal firing properties. Hybrid GEVI represent the latest advancement in voltage imaging. These indicators combine the optical properties of chemically synthesized VSD with genetic encoding of a membrane protein. Hybrid GEVI can operate via a voltage-dependent modulation of FRET (opsin-dye FRET, or fluorescent protein–dye FRET) or via chemical photo-induced electron transfer. The latter dyes require a synthetic counterpart to be delivered to the target cell, specifically a linker molecule that couples the chemically synthesized dye with a genetically encoded membrane protein (e.g., VoltageSpy GEVI [347]).

Class I and II GEVI provide rather dim signals due to their low sensitivity for which they require very intense light sources. Moreover, they suffer from unspecific tissue staining. In turn, these issues affect the SNR and the clear delimitation of the cellular contour. Hybrid GEVI promise to overcome these issues. Ongoing optimization of GEVI is increasingly providing novel tools for more accurate neurophysiological studies, e.g., probing multiple cell types simultaneously or resolving sub-cellular components like single synapses, and to increase the optical efficiency and SNR of these promising probes [348].

So far, voltage indicators have been used to study neuronal network dynamics in 2D models, such as primary neurons and human iPSC-derived neuronal cultures [332], in acute brain slices [349,350,351,352], organotypic brain slices [353], or in vivo [334]. To the best of our knowledge, no one has so far used them to probe network features in bioengineered brain tissue (be it scaffold-based 3D cultures, spheroids, or organoids). Nonetheless, their potential to be used with human iPSC-derived neurons [332] opens multiple possibilities for their deployment in BoC. Along with the development of novel engineered molecular constructs, voltage indicators, and particularly GEVI, and their combination with GECI, may represent a readily available all-optical approach to pinpoint non-invasively the input/output dynamics of bioengineered neuronal networks.

### 5.3. Optogenetics

Optogenetics combines optics and genetics to alter with light the electrical behavior of genetically engineered neurons and it permits precise and selective control of neuronal firing, as well as pre- and post-synaptic neuronal responses to neurotransmitter release, while, at the same time, targeting specific cell types. Here, we may distinguish two primary approaches: (i) *classic* optogenetics and (ii) chemical optogenetics. In classic optogenetics, exogenous light-sensitive ion channels, mostly of microbial origin, are genetically encoded into the target cell(s). Similar to voltage-dependent ion channels, light-sensitive ion channels are charge conducting transmembrane proteins, characterized by very short-latency activation. However, rather than being gated by changes in transmembrane potential, they are gated by light. In chemical optogenetics, native ion channels and receptors are genetically engineered to enable binding of a reversibly photoswitchable ligand. As the photoswitchable ligand is an externally applied compound, chemical optogenetics is also referred to as optogenetic pharmacology [354].

Classic optogenetics is a relatively young field that has been established at the beginning of 2000s, following the seminal works by P. Hegeman, G. Nagel and K. Deisseroth [355,356,357], which eventually led to the expression of channelrhodopsin (ChR) in neurons using genetic engineering techniques [358]. Opsins are the protagonist in classic optogenetics. They are photosensitive ion channels, initially described by C. Schilde in 1968 [359], which have now become a vast family. Ongoing research is indeed bringing the discovery of new naturally-occurring opsins, while genetic engineering is concurrently expanding the opsin family to include chimeric proteins. The most known and most commonly used naturally-occurring opsins are of microbial origin. Among these, three types are particularly relevant to optogenetics: the hyperpolarizing bacteriorhodopsin and halorhodopsin, which exert their action via proton efflux and chloride influx, respectively, and the depolarizing ChR1 and ChR2, which exert their action via cation-selective inward conductance. Thanks to the exponential progress in classic optogenetics, there is currently a plethora of available engineered and natural opsins that enable a high degree of experimental flexibility, including targeting selective cell sub-types such as interneuron sub-populations [360] or combining different optical approaches by exploiting different wavelength sensitivities of spectrally-shifted opsins, such as red-shifted opsins [361,362,363,364]. A detailed overview of the biophysical properties of available opsins is provided in [365,366].

Opsins are most commonly introduced in the model organism via viral expression systems leveraging lentivirus and adeno-associated virus vectors. Although the most common of these vectors carry ubiquitous or pan-neuronal promoters, some promoter fragments offer cell type-specificity, thereby making it possible to selectively target cell sub-populations in animals without using transgenic technologies. The latter remain in any case the best approach to restrict gene expression to specific neuronal subsets. Other routes include in utero electroporation for developmental targeting, circuit-specific targeting and conditional expression [367].

Chemical optogenetics is also a relatively young field, as the first description of its application dates to the beginning of the 2000s [368]. As opposed to classic optogenetics, which controls neurotransmitter release by the genetically-targeted cells, chemical optogenetics is particularly useful when the experimental protocol requires the direct activation or silencing of a specific ion channel or neurotransmitter receptor.

Portraits of this strategy are photoisomerizable tethered ligands (PTL) made specifically for cysteine-engineered native channels or receptors [369]. The latter are achieved by introducing a point mutation in the native channel or receptor protein via genetic engineering techniques. PTL offer the advantage of a modular structure, thanks to which it is possible to fine-tune their properties by individually addressing the design of each of their modules. The general PTL structure is composed of (i) reactant, which is the cysteine-reactive group mediating the covalent binding with the target protein, (ii) a photoisomerizable linker or photoswitch, which is the chemical group which either presents or removes the ligand from its binding site by changing its conformation upon exposure to light of specific wavelengths, (iii) ligand, which can be an ion channel pore blocker, or a neurotransmitter receptor agonist or antagonist. The photoswitch is the crucial chemical component of PTL. Several chemical groups can act as photoswitches, including spiropyrans, thioamids, and azobenzenes [369]. The latter are the most popular photoswitches since they can offer excellent photophysical properties as well as robust and efficient switching.

The first described genetically-targeted PTL-based system is the synthetic photoisomerizable azobenzene-regulated K^+^ channel (SPARK), which enabled, for the first time, direct silencing or resuming of action potential firing in genetically-targeted neurons, by directly exploiting, in an optogenetic approach, the ionic conductance mechanisms involved in the membrane repolarization phase of action potentials [368]. Subsequently, by exploiting a known single point mutation in the Shaker channel ion selectivity filter to increase its permeability to Na^+^ [370], the depolarizing (D)-SPARK was engineered to allow control of depolarization and action potential generation in target neurons [371]. PTL are being continuously developed, and today, in addition to controlling ion channels, chemical optogenetics can be used to control ionotropic glutamate receptors [372,373,374,375], metabotropic glutamate receptors [376], GABA-A receptors [377], and acetylcholine receptors [378,379] (see also [354]).

From the application standpoint, opsins and PTL requiring low light intensities can eliminate the need for bulky bench-top lasers and optical cables that far exceed the dimensions of a brain organoid and would not be suitable for integration within BoC platforms. In this regard, micro light emitting diodes (μLED) and/or laser diodes represent a solution for integrated electrophysiology and optogenetics applied to BoC, as they can be coupled to micron-scale multimode fibers placed directly on the MEA or silicon probe. The integration of μLED or laser diodes can be achieved primarily via two approaches: (i) monolithic integration of all optical and electrical components e.g., using photolithography techniques [380,381] and (ii) heterogeneous integration of a μLED assembly in the proximity of the sites for electrical recording by transfer printing [382,383,384]. In either case it is possible to finely control the distances between optical stimulation sites and recording electrodes (e.g., by photolithography), so as to perform simultaneous optogenetic modulation and electrical monitoring of small neuronal ensembles down to the single neuron [380,381,385].

So far, only a few studies have made use of optogenetic approaches to probe brain organoid function, all of which are based on classic optogenetics. These include retinal organoids [386], cortico-striatal assembloids [387] and motor neurons coupled to muscle cells in a model of amyotrophic lateral sclerosis [388]. Noteworthy, whereas in [386] retinal cells needed to be genetically engineered, in [142] whole-brain organoids exhibited a sub-population of naturally photosensitive cells. Finally, it is worth mentioning the study by Mansour and colleagues [138] in which optogenetics was used to assess the functional integration of a grafted organoid within the host brain.

In terms of safety, photoswitches raise some concerns for their translational value due to the poor solubility, metabolic stability and potential cytotoxicity of some of them, especially azobenzenes and their derivatives, which may yield potentially toxic metabolites [389]. In this regard, more research is needed for an in-depth assessment of the toxicity of these compounds, as well as to design other molecules that can be suitable for safe use in humans. At variance, it has been recently shown that microbial opsins do not exert any significant cytotoxic effect in human iPSC-derived organoids [386], letting foresee their safe deployment in the clinical setting.

It is important to emphasize that the potential of optogenetics goes well beyond modulating the electrical behavior of neurons. This technique is proving a powerful tool in the era of stem cell biotechnology and brain tissue bioengineering [390]. Classic optogenetics has also proved useful to regulate gene expression [391], guide stem cell fate during development [392,393], study and modulate signaling pathways involved in embryogenesis [394,395]. Chemical optogenetics has been used to modulate immune responses [396] and intracellular signaling pathways, such as cAMP synthesis [397], GTPase-mediated cytoskeletal function enabling photocontrol of cell motility and shape [398], and the versatile tunable light-controlled protein tags enabling regulation of a variety of signaling pathways [399]. Highly relevant to brain tissue bioengineering is the emergence of photosensitive transcription factors [400], which open the prospective of manipulating growth and differentiation of multicellular systems with cell-type-specific, spatially-restricted and temporally-controlled gene expression.

Altogether, the great versatility of optogenetics can enable both electrical and metabolic control over brain organoids. As brain developmental processes determine brain architecture, and since structure and function are tightly inter-related, the integration of optogenetic tools in BoC platforms will therefore provide a unified strategy for finely controlled brain organoid bioengineering, from the cellular, architectural and functional standpoints.

## 6. Conclusions and Future Technology Perspectives

In this review we have portrayed the progression of BoC biotechnology and the electrophysiology read-out tools that can benefit the BoC field, from their origins to the present state-of-the-art. Currently, the BoC paradigm is primarily considered for two applications: (i) animal testing replacement for drug discovery and screening, and for personalized medicine [3,401], and (ii) brain development, function and dysfunction studies [2,3,124,131]. However, as outlined below, the possibilities of BoC biotechnology go well beyond these commonly considered applications, and its full potential is yet to be achieved.

BoC may provide unprecedented strategies for regenerative medicine, as heralded by the foundational works from the labs of Takeuchi [118], Gage [138] and Liu [139], demonstrating the feasibility of grafting bioengineered brain tissue in the rodent brain. Nonetheless, we are still far from the safe implementation of this paradigm. First, tissue engineering strategies have so far been able to achieve in brain organoids (and the like) an early stage of maturation only, which resembles that of the embryonic or fetal brain, whereas structural and functional features of juvenile or adult brain maturation are still missing. This drawback likely reflects a gap of knowledge in embryology along with the large technical difficulties in maintaining very long-term cell cultures as would be needed for organoid maturation to the adult phenotype. The required biological know-how is not yet available and will require more knowledge from all sectors of neuroscience, entailing a large and long effort to be filled. Second, an in-depth and long-term assessment of the functional consequences of bioengineered brain tissue grafting in vivo has not been performed yet. Third, in vitro systematic studies for the functional characterization of bioengineered brain tissue are still missing, while only scattered information is available.

MEMS–microfluidics platforms for BoC may allow the pinpointing and deployment of key intrinsic and extrinsic microenvironmental cues involved in brain development, thereby enabling the achievement of more realistic biological substrates, toward safe in vivo grafting. However, one missing, yet key element within BoC platforms is the by-default integration of electrophysiology tools to evaluate the function as well as the structure-function relationship of the bioengineered brain tissue. Along with electrical read-out, BoC platforms should enable probing and modulation of the tissue input/output features. This can be attained by electrical stimulation, by optogenetic approaches (see §5.3) or by localized delivery of specific molecules, e.g., via microfluidics MEAs [194]. A step further would be implementing closed-loop paradigms to these probing strategies: being based on real-time feed-back from the electrical activity generated by the bioengineered brain tissue, closed-loop paradigms would offer vast possibilities to dynamically interact with the tissue at all levels (molecular, sub-cellular, cellular, sub-network, or network-wide). In addition, the integrated sensing of electrochemical signals [402] would allow correlating the neurotransmitter release with electrical signal propagation within the neuronal network, opening the possibility for more complex closed-loop paradigms based on multi-parameter sensing. Ultimately, closed-loop approaches foresee an adaptive BoC platform, wherein the bioengineered brain tissue could be coupled to artificial neuron-like devices (i.e., neuromorphic systems) for fully autonomous functional biohybrids capable of self-reconfiguration [402,403]. However, at the present time, there is no platform offering these features in a fully integrated single chip.

The growing demand for increasingly complex miniaturized implantable bio-MEMS lets foresee the possibility of their self-standing incorporation within bioengineered brain tissue to probe and modulate its function. Such outstanding advancement can be achieved by the integration of biosensors and actuators, drug delivery systems, and μLED for on-chip optogenetics, data telemetry for remote monitoring of electrical activity, and power autonomy to bypass the limitations of device tethering. In this regard, the field has already showcased its moves in this direction (see §4.3.3), heralding the possibility of functional biohybrid constructs that may represent a major breakthrough in regenerative medicine to cure brain disorders, as they may enable previously unwitnessed control (and, therefore, safety) over brain regeneration processes.

## Figures and Tables

**Table 1:** Biomaterials for scaffold-based 3D cultures ^1^.

Biomaterial Type	Biomaterial Name	Scaffold Type
**Synthetic**	polystyrene, poly-L-lactic acid [78]	porous solid
	tyrosine-derived polycarbonate [79]	electrospun fibers
	graphene [72]	nanostructured
	PDMS-graphene [80]	
	CNT-graphene [81]	
	PDMS-CNT [73]	
	PAA-graphene [82]	
	glass [83]	microstructured
	PEG [76,84]	hydrogel
**Semi-synthetic**	transglutaminase cross-linked hyaluronic acid [85,86]	hydrogel
	methacrylamide-chitosan [87]	
	carboxymethyl-chitosan [88] carboxylmethyl-chitosan – alginate – agarose [89]	
	methylcellulose–laminin [90]	
	(PEG)ylated fibrinogen [91]	
**Natural – Vegetal**	cellulose paper [92,93]	porous solid
	alginate [94,95,96,97]	hydrogel
**Natural – Animal**	chitosan [98] matrigel [99] collagen [100] silk fibroin [101] silk fibroin – collagen [102] silk fibroin – collagen – decellularized porcine brain tissue [103] decellularized human fat tissue [104]	hydrogel

^1^ CNT: carbon nanotubes. PAA: polyacrylamide. PEG: polyethylene glycol. PDMS: poly-dimethylsiloxane.

**Table 2:** Electrophysiology techniques used for functional studies in different organoid types and preparations.

Electrophysiology Technique	Organoid Type	Sample Processing	References Within This Review
Patch-Clamp	Region-specific (Cortical)	Intact	[148,158]
Patch-Clamp	Region-specific (Cortico–hippocampal)	Dissociated	[133]
Patch-Clamp	Region-specific (Cortical)	Slice	[52,159]
Patch-Clamp	Non-region-specific	Slice	[141,149,156]
Planar MEA	Non-region-specific	Slice	[149]
Planar MEA	Region-specific (Cortical)	Intact	[148,158]
Planar MEA	Non-region-specific	Intact	[160,161]
Silicon Probe	Non-region-specific	Intact	[142]
Silicon Probe	Region-specific (Cortical)	Slice	[159]
3D MEA	Non-region-specific	Slice	[141]
